# A Low-Correlation Resting State of the Striatum during Cortical Avalanches and Its Role in Movement Suppression

**DOI:** 10.1371/journal.pbio.1002582

**Published:** 2016-12-06

**Authors:** Andreas Klaus, Dietmar Plenz

**Affiliations:** Section on Critical Brain Dynamics, National Institute of Mental Health, Bethesda, Maryland, United States of America; University of Minnesota, UNITED STATES

## Abstract

During quiet resting behavior, involuntary movements are suppressed. Such movement control is attributed to cortico-basal ganglia loops, yet population dynamics within these loops during resting and their relation to involuntary movements are not well characterized. Here, we show by recording cortical and striatal ongoing population activity in awake rats during quiet resting that intrastriatal inhibition maintains a low-correlation striatal resting state in the presence of cortical neuronal avalanches. Involuntary movements arise from disturbed striatal resting activity through two different population dynamics. Nonselectively reducing intrastriatal γ-aminobutyric acid (GABA) receptor-A inhibition synchronizes striatal dynamics, leading to involuntary movements at low rate. In contrast, reducing striatal interneuron (IN)-mediated inhibition maintains decorrelation and induces intermittent involuntary movements at high rate. This latter scenario was highly effective in modulating cortical dynamics at a subsecond timescale. To distinguish intrastriatal processing from loop dynamics, cortex-striatum-midbrain cultures, which lack feedback to cortex, were used. Cortical avalanches in vitro were accompanied by low-correlated resting activity in the striatum and nonselective reduction in striatal inhibition synchronized striatal neurons similar to in vivo. Importantly, reduction of inhibition from striatal INs maintained low correlations in the striatum while reorganizing functional connectivities among striatal neurons. Our results demonstrate the importance of two major striatal microcircuits in distinctly regulating striatal and cortical resting state dynamics. These findings suggest that specific functional connectivities of the striatum that are maintained by local inhibition are important in movement control.

## Abstract 2

Why don’t neuronal “avalanches” in resting-state cortex cause involuntary movements? This study shows that a low-correlation striatal resting state suppresses such movements and explores mechanisms that disrupt this inhibition.

## Author Summary

Even in the absence of apparent motor output, the brain produces a rich repertoire of neuronal activity patterns known as “resting state” activity. In the outer layer of the cortex, resting state patterns emerge as neuronal avalanches, precisely scale-invariant spatiotemporal bursts that often engage large populations of neurons. Little is known about how the brain suppresses involuntary movements during such activity. Here, we show that the striatum, which is part of the cortex-basal ganglia loop, maintains a low-correlation state during resting activity. By using a combination of in vivo and in vitro approaches with pharmacological manipulations, we demonstrate that the precise configuration of this low-correlation state effectively contributes to involuntary movements. Nonselective blockade of intra-striatal inhibition abolished the low-correlation striatal resting state, barely affected cortical avalanches, and led to involuntary movements at low rate. In contrast, selectively reducing striatal interneuron inhibition strongly affected cortical avalanches and triggered involuntary movements at high rate while maintaining a relatively decorrelated striatal resting state. Our results demonstrate the importance of different inhibitory striatal circuits in the suppression of involuntary movements and suggest that the precise spatiotemporal configuration of striatal activity plays an active role in the regulation of cortical resting state activity and motor control.

## Introduction

In the absence of specific sensory input or motor output, the brain nevertheless is highly active. In the cortex, such resting activity exhibits long-range spatial and temporal correlations [1–3], with intermittent neuronal bursts described by power laws and defined as neuronal avalanches [4]. Neuronal avalanches have been identified in spontaneous activity in vitro in isolated cortex preparations [4–6] as well as in vivo in rodents [7–9], nonhuman primates [10–13] and humans [2,14,15], suggesting that, during resting, the cortex resides close to a critical state [16,17] at which numerous aspects of information processing are optimized [18].

The scale-free nature of cortical avalanches implies maximal variability in size and synchrony of neuronal events [19,20]. When monitored in motor cortical areas, avalanches unfold without the presence of apparent movements [10,13], raising the question why even large avalanches during resting do not translate into sporadic or involuntary motor outputs. Here, we study this question in the context of forebrain loops that encompass cortex and basal ganglia and that are considered crucial for the initiation of voluntary as well as suppression of involuntary movements [21–25].

The main entry point from cortex to the basal ganglia is the striatum, which consists of more than 95% of γ-aminobutyric acid (GABA)-releasing spiny projection neurons (SPNs) and a small percentage of GABAergic interneurons (INs), particularly parvalbumin-positive, fast-spiking INs [26,27]. Although changes in intrastriatal inhibition have long been identified to lie at the core of many movement disorders (e.g., [23,25]), the distinct roles of SPNs and INs remain unclear. SPNs form a sparse network of inhibitory recurrent connections with each other [28–30], which theory and simulations suggest support competitive dynamics [31,32] that decorrelate networks [33]. In contrast, striatal fast-spiking INs provide a dense network of perisomatic inhibitory connections on SPNs, typically interpreted as cortical feedforward inhibition of SPNs [34–37]. Reducing striatal fast-spiking neuron activity induces involuntary movements in rodents [38], in line with a reduced number of those neurons in humans suffering from Tourette syndrome [39,40]. However, how inhibition in striatal microcircuits relates to cortical avalanche dynamics at rest and suppresses involuntary movements is unclear.

Here, we demonstrate in awake rats during quiet resting that cortical activity organizes as neuronal avalanches, whereas the striatum actively maintains a low-correlation state. Involuntary movements emerge from this dynamical profile through two distinct mechanisms. During nonselective reduction of inhibition in the striatum, movements emerged at low rate with little change in cortical avalanches but large increase in striatal synchrony. In contrast, when reducing inhibition from striatal INs only, movements emerged at high rate with corresponding large changes in cortical avalanches yet small change in relative striatal synchrony. In both scenarios, involuntary movements correlated with striatal and cortical bursts. To distinguish intrastriatal processing from loop dynamics, cortex-striatum-midbrain cultures, which lack feedback to cortex, were employed. Cortical avalanches in vitro were accompanied by low-correlated resting activity in the striatum and nonselective reduction in striatal inhibition synchronized striatal neurons, similar to in vivo. Importantly, reduction of inhibition from striatal INs maintained low correlations in the striatum while reorganizing functional connectivities among striatal neurons. Our findings demonstrate the importance of two major striatal microcircuits in distinctly regulating striatal and cortical resting state dynamics. We suggest that specific functional connectivities of the striatum that are maintained by local inhibition are important in movement control.

## Results

### Two Striatal Disinhibition Models That Induce Involuntary Movements In Vivo

**Fig 1:**
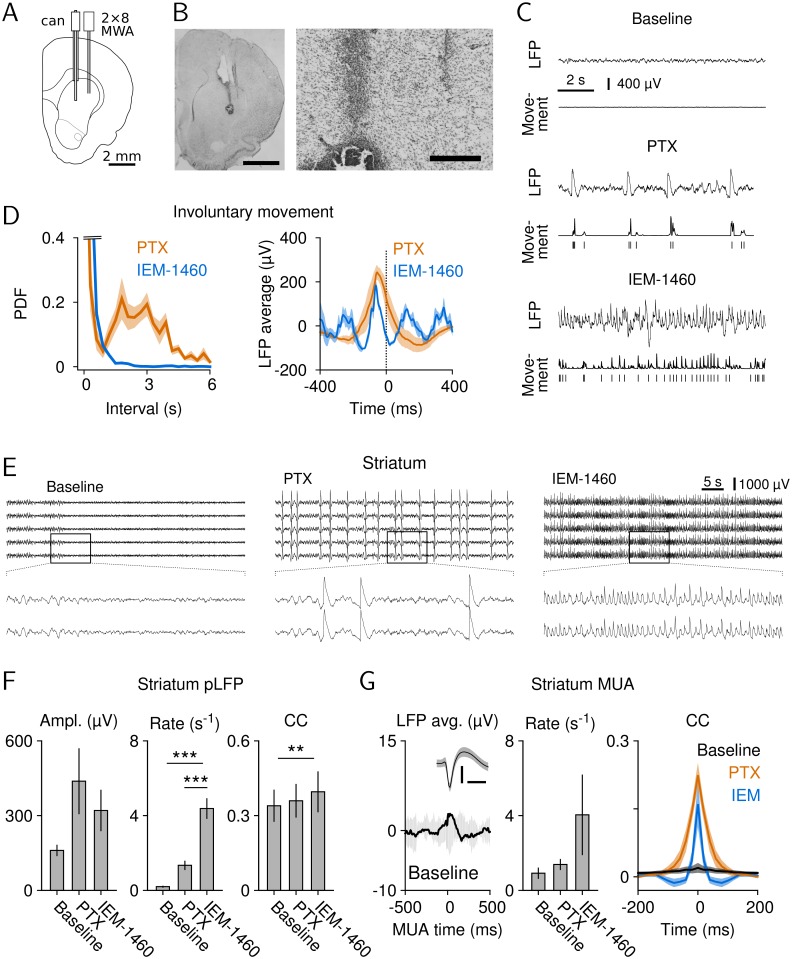
Local striatal disinhibition induces involuntary movements synchronized with striatal burst activity. (A) Depiction of the location of cannula (can) and 2×8-channel MWA in the dorsolateral striatum. (B) Nissl stain of a coronal section showing cannula and wire tracks (left: entire hemisphere, scale bar: 2 mm; right: detailed view, scale bar: 250 μm). Note the small lesion restricted to the injection site. (C) Example traces of LFP (top) and front-paw movements (bottom, arbitrary units) are shown for all three conditions. For PTX and IEM-1460, involuntary movements were extracted by thresholding (2 standard deviations [SD]), as indicated by the marks below the movement signal. (D) Left: probability density function (PDF) of intervals between involuntary movements in the contralateral front paw or neck after blockade of striatal inhibition (PTX, 1 mM, *n* = 8) or AMPA-mediated input to striatal INs (IEM-1460, 5 mM, *n* = 7). Right: involuntary movement-triggered LFP average (*n* = 3). (E) LFPs change during striatal disinhibition (top: 5 out of 16 channels are shown; bottom: detailed 10-s view, as indicated by the rectangle in the top panel). (F) Amplitude, rate, and cross-correlation (CC) of pLFPs under all conditions (baseline: *n* = 8; PTX: *n* = 7; IEM-1460: *n* = 7; rANOVA, **p < 0.01, ***p < 0.001). pLFP threshold: 3 SD. Bin size: 4 ms. Number of electrode pairs for CC: baseline, 886 pairs total; PTX and IEM-1460, each 781 pairs total. (G) Left: MUA-triggered LFP average showing weak coupling between LFP and MUA (black: average of *n* = 5 rats, gray: ±3 SD of MUA-shuffled LFP average). Left, inset: Average MUA (scale bars: 500 μs, 10 μV). Middle: Rate of MUA reflects changes in pLFPs. Right: MUA CC as a function of time lag for all three conditions: baseline (*n* = 5 rats, 175 pairs total), PTX (*n* = 4 rats, 226 pairs total), IEM-1460 (*n* = 4 rats, 129 pairs total). Data for this figure are in S1 Data.

To study striatal resting activity during cortical avalanches and its change during involuntary movements, we exploited two dyskinesia models in rat. Involuntary movements in vivo have been induced either by nonselective reduction of intrastriatal inhibition using the GABA_A_-antagonist picrotoxin (PTX) [41–45] or selective reduction of striatal IN-mediated inhibition [38] using IEM-1460 [46,47]. Accordingly, we chronically implanted a cannula guide for local drug injection combined with a 16-channel microwire array (MWA) into the dorsal striatum (Fig 1A and 1B; S1 Fig). Ongoing striatal local field potentials (LFPs) and multi-unit activity (MUA) were recorded before, during, and after local drug infusion in unrestrained awake rats not involved in any particular task. PTX (1 mM; *n* = 8 rats) induced stereotypical movements at low rate (0.58 ± 0.06 s^-1^) in the contralateral front paw and/or neck region (Fig 1C and 1D, left; S1 Movie). In contrast, IEM-1460 (5 mM) induced more variable, intermittent movements at ~6 times higher rate (3.45 ± 0.44 s^-1^; S2 Movie; *n* = 7 rats; paired *t* test, *t*(10) = –7.9, *p* < 0.001; PTX versus IEM) in the contralateral front paw (Fig 1C and 1D, left; S2 and S3 Figs). Involuntary movements correlated with positive LFP (pLFP) deflections in the striatum (Fig 1C and 1D, right), which mirrored the significant increase in rate for IEM-1460- over PTX-induced movements (Fig 1E and 1F, middle; rANOVA, F(2,12) = 51.84, *p* < 0.001). Importantly, the increased pLFP rate under IEM-1460 was not dose dependent, as a ten times lower concentration of IEM-1460 induced pLFPs at a similar rate as 5 mM (S3 Fig; pLFP rate at 0.5 mM: 3.96 ± 0.76 s^-1^; 5 mM: 4.38 ± 0.53 s^-1^).

The change in pLFP rate was not paralleled by a corresponding change in cross-correlation (CC) between pLFPs, which was found to be relatively high at baseline and increased only weakly under PTX or IEM-1460 (Fig 1F, right; 4 ms bin size; rANOVA, F(2,12) = 10.13, *p* = 0.003; 1.16-fold increase). Because pLFPs could largely reflect synaptic input to the striatum, we additionally analyzed striatal MUA, which more directly reflects intrastriatal processing. Indeed, striatal MUA showed an increase in rate as well as an order of magnitude increase in spatial correlations for dyskinetic conditions. At baseline, spatial MUA correlations were low (r = 0.02 ± 0.01; *n* = 5 rats, 20 ms bin size) and increased 8-fold for IEM-1460 and even 11-fold for PTX (Fig 1G, right; IEM-1460: r = 0.16 ± 0.05; *n* = 4 rats; PTX: r = 0.22 ± 0.03, *n* = 4 rats; rANOVA, F(2,6) = 10.23, *p* = 0.012; baseline versus PTX: *p* = 0.013, baseline versus IEM-1460: *p* = 0.069, Bonferroni-corrected). Similarly, the temporal correlation between MUA was also wider for PTX than IEM (Fig 1G, right; PTX: 60.0 ± 6.63 ms; IEM-1460: 34.3 ± 9.5 ms; half-width in the CC function). The dissociation between pLFP- and MUA-based measures is supported by the weak correlation between striatal MUA and the LFP under baseline conditions (Fig 1G, left; S4 Fig). Our findings so far suggest that striatal activity changes from a weakly correlated state during resting to a more correlated state under PTX- and IEM-1460-induced dyskinesia, with IN-mediated disinhibition causing involuntary movements at higher rate compared to nonselective striatal disinhibition.

### Reduction in Striatal IN-Mediated Inhibition Induces Involuntary Movements with Little Change in Relative Striatal Synchrony

Changes in striatal MUA correlation could still reflect changes to striatal input rather than differences in local striatal processing. Specifically, the recruitment of cortico-basal ganglia loops during involuntary movements is supported by early reports on interrupting involuntary movements through cortical cooling in rodents and the emergence of synchronized cortical and striatal LFP deflections before movement onset [48].

**Fig 2:**
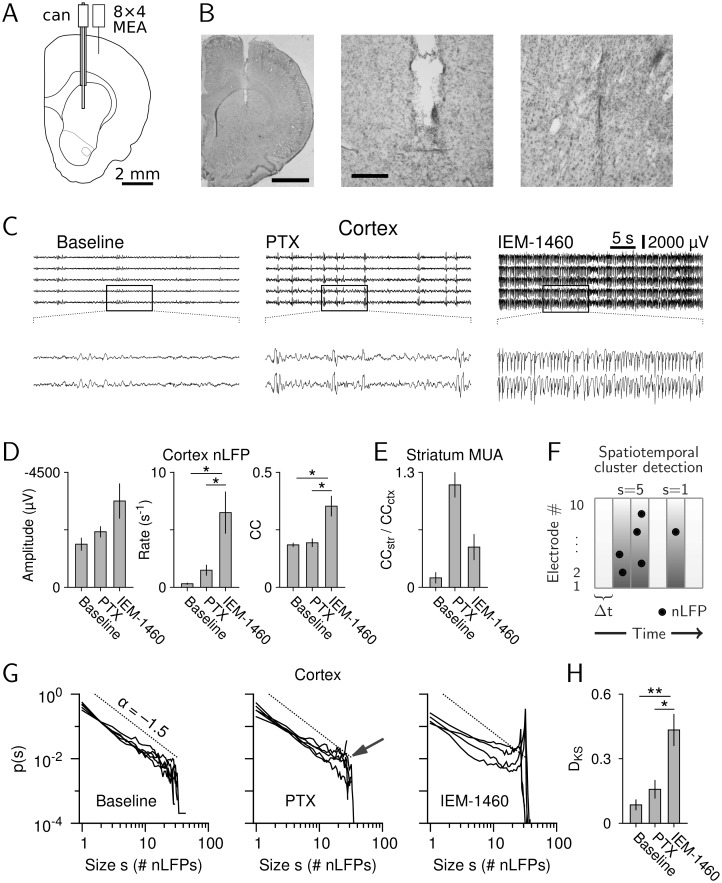
Intrastriatal disinhibition recruits cortico-basal ganglia loops. (A) Depiction of the location of cannula (can) for local striatal drug injection, and 8×4-channel microelectrode array (MEA) implanted in superficial cortical layers. (B) Nissl stain of a coronal section showing cannula and electrode array tracks (left: entire hemisphere, scale bar: 2 mm; middle and right: detailed views of cannula and array location, respectively; scale bar: 250 μm). (C) Examples of cortical LFP during baseline, PTX (1 mM), and IEM-1460 (5 mM). Top: 5 out of 32 channels are shown. Bottom: detailed 10-s view as indicated by the rectangle in the top panel. (D) Amplitude, rate, and CC of nLFPs under all conditions (baseline: *n* = 5 rats; PTX: *n* = 5 rats; IEM-1460: *n* = 4 rats; nLFP threshold: −2.5 SD; bin size: 4 ms; *p < 0.05). (E) Relative striatal synchrony, that is, striatal MUA CC normalized by cortical nLFP CC. (F) Illustration of detection algorithm for spatiotemporal nLFP clusters. The size, *s*, of a cluster is the number of nLFPs in the cluster. (G) Cluster size distributions for all three conditions. Arrow indicates slight increase of probability of larger clusters under PTX. (H) Kolmogorov–Smirnov distance (D_KS_) between experimental distributions and power law with exponent −1.5 shows a significant deviation from avalanche dynamics for IEM-1460 (*p < 0.05, **p < 0.01). Data for this figure are in S2 Data.

Indeed, when recording ongoing LFP and MUA activity in cortex from up to 32 electrodes while repeating our local infusion of PTX or IEM-1460 into the striatum (Fig 2A and 2B), the cortical LFP was found to change similar to the striatal LFP (Fig 2C). In particular, the rate of negative LFP (nLFP) deflections in cortex was significantly higher during IEM-1460 than PTX (Fig 2D, middle; see also Fig 1F). In contrast, spatial correlations in cortex were markedly increased for IEM-1460 but not for PTX compared to baseline (Fig 2D, right; 4 ms bin size; rANOVA, F(2,6) = 12.0, *p* = 0.008; baseline versus IEM-1460: *p* = 0.014; PTX versus IEM-1460: *p* = 0.019, Bonferroni-corrected), which differed from what we found for the striatum (see also Fig 1F, right). Given that cortical MUA strongly correlated with cortical nLFPs during all conditions (S5 Fig), the CC for cortical MUA was also found to be the largest for IEM-1460 (baseline: 0.02 ± 0.0, *n* = 5; PTX: 0.07 ± 0.03, *n* = 5; IEM-1460: 0.12 ± 0.05, *n* = 2).

This increase in cortical synchronization for IEM-1460 compared to PTX suggests that synchronization of striatal activity under IEM-1460 might be largely explained by changes in cortical activity. To compare the striatal change in synchrony relative to that in cortex, we normalized the average spatial correlation in the striatum by that found in cortex. Indeed, PTX-induced movements revealed a strong increase in relative striatal synchrony, whereas IEM-1460–induced movements emerged from relatively decorrelated striatal conditions (Fig 2E). This decorrelated striatal state under normal resting conditions and during IEM-1460–induced movements was confirmed whether using cortical LFP or cortical MUA, which strongly correlated with cortical LFP during all conditions (S5 Fig). Taken together, these results establish two vastly different population scenarios for striatal induction of involuntary movements—a nonselective disinhibition, which induces movements at low rate in face of large relative striatal synchrony, and a selective reduction of IN-mediated inhibition, which induces movements at high rate with modest changes in relative striatal synchrony.

### Reduction in Striatal IN-Mediated Inhibition Effectively Deviates Cortical Resting Activity from Avalanche Dynamics

We next demonstrated that the observed changes in striatal and cortical activity indeed arise from a resting state in cortex that organizes in the form of neuronal avalanches, and that, compared to PTX, IEM-1460–induced involuntary movements are more effective in introducing deviations from avalanche dynamics. Neuronal avalanches reflect spatiotemporal clusters of activity, which, besides pairwise correlations, also contain significant higher-order correlations that establish precise scale-invariant dynamics in space and time [12,13]. Cortical avalanches have been described in local populations of pyramidal neurons [7,9] and at the mesoscopic scale using nLFPs [4,8] as well as in humans using magnetoencephalography and functional magnetic resonance imaging [1,2,14,15]. In cortex, nLFPs are associated with increased firing in local synchronized neuronal populations [8,10,49]. We therefore used the nLFP (S5 Fig) to measure spatiotemporal activity clusters and quantify cortical dynamics. Fig 2F illustrates the definition of spatiotemporal avalanches using a given threshold for detection of nLFPs (black dots) and bin size, Δt, for concatenation of successive nLFPs into spatiotemporal clusters (adjacent dark gray time bins). In line with previous reports on ongoing activity in vivo [8], spatiotemporal clusters of cortical nLFPs during baseline distributed in size according to a power law with exponent α = –1.45 ± 0.08 and cut off at array size close to 32, the defining characteristics of avalanche dynamics ([50]; Fig 2G, baseline; threshold: −2.5 standard deviation (SD), Δt = 4 ms; *n* = 5 rats; power law versus exponential: log-likelihood ratio (LLR) = 169.1 − 2738.1, all *p* < 0.001 in favor of power law, see Materials and Methods). The power law barely changed during PTX (Fig 2G, middle; same threshold as for baseline), whereas IEM-1460 increased the probability of large cortical clusters significantly compared to baseline and PTX (Fig 2G, right), as measured by the Kolmogorov–Smirnov (KS) distance (D_KS_), which here quantifies the deviation from a power law with exponent α = −1.5 (Fig 2H, rANOVA, F(2,6) = 16.92, *p* = 0.003; baseline versus IEM-1460: *p* = 0.004, PTX versus IEM-1460: *p* = 0.019, Bonferroni-corrected). In line with the observed increase in nLFP frequency, the rate of spatiotemporal clusters increased during both drug conditions and was highest under IEM-1460 (baseline: 1.93 ± 0.75 s^-1^, PTX: 7.27 ± 4.72 s^-1^, IEM-1460: 12.3 ± 3.66 s^-1^). Importantly, the average duration of spatiotemporal clusters was less than 10 ms under all conditions and thus approximately one order of magnitude shorter than the time between clusters, indicating that the increased probability of larger clusters under IEM-1460 did not result from coalescing clusters due to the chosen bin time, Δt.

In summary, a striatal resting state, in which IN-mediated inhibition is reduced, is highly effective in entraining cortical dynamics away from neuronal avalanches.

### Low-Correlation Striatal Resting State in Response to Cortical Avalanches In Vitro

**Fig 3:**
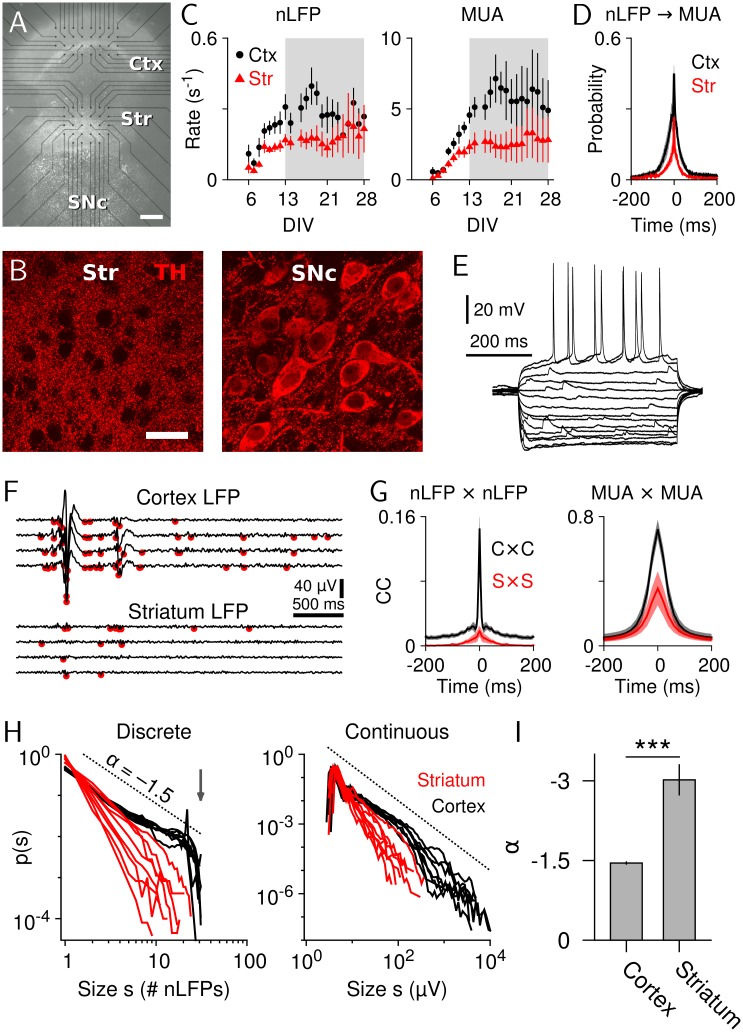
A decorrelated resting state of the striatum emerges in the presence of neuronal avalanches in cortex (open-loop in vitro model). (A) Organotypic culture after 14 DIV. Ctx: cortex, Str: striatum, SNc: substantia nigra pars compacta. Scale bar: 500 μm. (B) Left: TH-positive fibers in the striatum at 14 DIV (5 μm maximum intensity z-projection) show a dense innervation (scale bar: 30 μm). Right: TH-stain of dopaminergic cells in the substantia nigra pars compacta (0.8 μm optical slice; same culture and scaling as in left panel). (C) Average rate of nLFPs (left) and MUA (right) in cortex (*n* = 4 − 6) and striatum (*n* = 5 − 7). Subsequent experiments were done between 13 and 28 DIV, indicated by the gray shaded area. (D) nLFP-triggered MUA at 14 DIV (cortex: *n* = 5 cultures; striatum: *n* = 6 cultures). (E) Voltage responses of a SPN to somatic current step injections (−200 to 80 pA; step size, 20 pA). (F) Example LFP and detection of nLFP peaks (red dots, threshold: –4.5 SD). (G) Average CC for nLFPs (left panel, 4 ms bin size) and MUA (right panel, 20 ms bin size) in cortex and striatum (*n* = 6 cultures). (H) Left: discrete cluster size distributions of cortical neuronal avalanches (black; broken line: visual guide to a power law with slope –1.5) and striatal clusters (red; *n* = 8 cultures). Vertical arrow indicates avalanche cutoff due to system size for the cortical sub-array (31 electrodes). Right: corresponding continuous cluster size distributions. (I) Average power-law exponent (***p < 0.001, *n* = 8). Data for this figure are in S3 Data. The data in panels A, F, and H have previously been published in [55].

**Fig 4:**
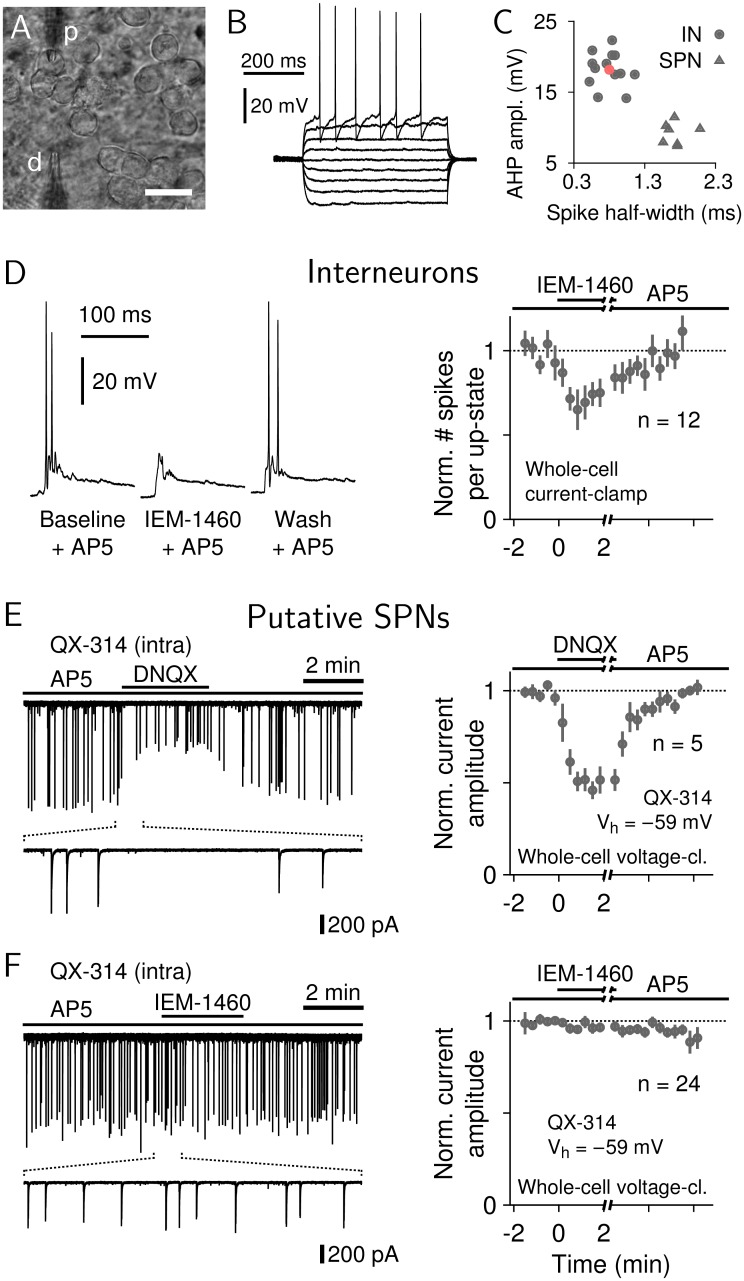
IEM-1460 selectively reduces firing of striatal INs in vitro. (A) Bright-field micrograph of a patched putative SPN illustrating the setup of local drug micro-application (p: patch pipette, d: drug pipette; scale bar: 20 μm). (B) Voltage responses of an electrophysiologically identified IN. (C) Spike parameters separate INs from SPNs in current-clamp mode (red dot indicates the neuron in B). (D) IN response to IEM-1460. Left: spontaneous up-states in an IN during baseline, 500 μM IEM-1460, and washout. All recordings were performed in the presence of 100 μM AP5 to block NMDA-receptor mediated currents. Right: average reduction of IN spiking under IEM-1460 (*n* = 12 INs). (E) Response of putative SPNs to the AMPA antagonist DNQX. Left: spontaneous AMPA-mediated up-state currents were reduced by 50 μM DNQX. To block active sodium currents, patch pipettes contained, in addition, 5 μM QX-314. Holding voltage: V_h_ = −59 mV. Right: average AMPA-currents in response to DNQX are strongly reduced in putative SPNs (*n* = 5), demonstrating that AMPA inputs to the patched neurons could be reduced in this patch configuration. (F) SPN response to IEM-1460. Left: spontaneous AMPA-mediated up-state currents in a putative SPN during baseline, 500 μM IEM-1460, and washout. Right: average AMPA-current amplitudes in putative SPNs (*n* = 24) do not change in response to IEM-1460. Data for this figure are in S4 Data.

In order to dissociate changes in striatal dynamics due to intrastriatal processing versus cortico-basal ganglia-thalamic loops, we next studied striatal responses to cortical avalanches in organotypic cortex-striatum-midbrain cultures, which lack striatal feedback to cortex [51,52]. Cultures were grown on custom planar microelectrode arrays (MEAs) with two electrode fields, allowing for simultaneous recording from cortex (8×4 electrodes) and striatum (6×5 electrodes) (Fig 3A). Recordings were performed between 13 to 28 days in vitro (DIV) when the striatum was innervated by corticostriatal projection neurons [53] and densely innervated by tyrosine-hydroxylase (TH)-positive fibers (Fig 3B, left) originating from substantia nigra neurons of the midbrain culture (Fig 3B, right; 175 ± 33 TH-positive neurons, range: 37–385; *n* = 11 cultures; [51]). During that period, cortical and striatal population activities were highest (Fig 3C) [54], showed stable activity profiles (S6 Fig), and electrophysiological properties of striatal neurons had matured appropriately (Figs 3E and 4B).

This open-loop in vitro system confirmed our in vivo finding that cortical neuronal avalanches are accompanied by low-correlated periods in striatal activity. First, nLFP amplitudes, which correlate with MUA activity (Fig 3D), as well as spatial correlations between nLFP or MUA activity were smaller in the striatum compared to cortex (Fig 3F and 3G). Second, spatiotemporal nLFP clusters in cortex revealed avalanche signatures, i.e., a power law in cluster size distribution with exponent α close to −1.5 (Fig 3H, black, discrete: *n* = 8, power law versus exponential: LLR = 1,564–27,090, all *p* < 0.001 in favor of power law; α = −1.47 ± 0.02, [4]). In contrast, striatal nLFP cluster size distributions, although consistent with a power law distribution (Fig 3H, red; *n* = 8, LLR = 123–3,225, all *p* < 0.01; α = −3.04 ± 0.27), showed a more negative exponent (Fig 3I; paired *t* test, *t*(7) = −5.9, *p* < 0.001); that is, the probability of large nLFP clusters was lower in striatum compared to cortex, in line with our finding of low spatial correlations in the striatum in vivo during resting activity. A similar relationship was observed when defining cluster size as the absolute sum of nLFP amplitudes (Fig 3H, continuous). The difference between cortical and striatal cluster size distributions was of dynamical nature because it was significantly reduced by bath application of PTX (4 μM; D_KS_ between cortical and striatal cluster size distributions, *n* = 8, rANOVA, F(2,14) = 11.67, *p* = 0.001, S7 Fig).

Our open-loop in vitro model confirms our in vivo finding that resting state activity in the form of cortical avalanches is associated with a low-correlation resting state in the striatum.

### PTX but Not IEM-1460 Amplifies and Synchronizes Striatal Responses to Cortical Input

To further study the differential effects of PTX and IEM-1460 on striatal dynamics observed in vivo, we first confirmed that IEM-1460 selectively suppressed firing in striatal INs in our in vitro system. Whole-cell current-clamp recordings (Fig 4A) of electrophysiologically identified INs (Fig 4B and 4C) showed that spontaneous action potential firing was significantly reduced in response to local application of 500 μM IEM-1460 (Fig 4D, *t* test, *t*(12) = 5.9, *p* < 0.001). To confirm that IEM-1460 did not affect AMPA-mediated excitatory postsynaptic currents in SPNs, we recorded spontaneous up-state currents in putative SPNs in the presence of QX-314 (5 μM, intracellular) and AP5 (100 μM, bath application) to block active sodium currents and N-methyl-D-aspartate (NMDA) receptors, respectively. To minimize inhibitory postsynaptic currents, voltage-clamp recordings were performed at the estimated GABA reversal potential, V_h_ = −59 mV. As expected, local ejection of the selective AMPA receptor antagonist DNQX significantly reduced up-state currents in all putative SPNs (Fig 4E, *t* test, *t*(12) = 4.6, *p* < 0.001). In contrast, local ejection of IEM-1460 did not significantly change the average peak amplitude of spontaneous compound postsynaptic currents in putative SPNs (Fig 4F, *t* test, *t*(12) = 0.3, *p* = 0.75). Taken together, these results show that IEM-1460 selectively reduces spontaneous firing in striatal INs without altering AMPA-mediated inputs to SPNs, in line with a previous study [38].

**Fig 5:**
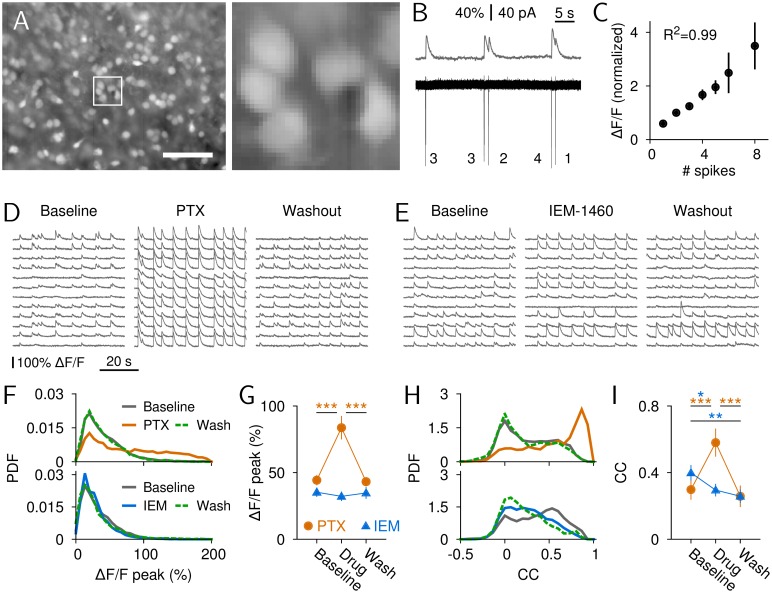
Increase in striatal response amplitude and correlation under PTX but not IEM-1460 in the open-loop condition. (A) Fluorescence image of striatal neurons loaded with OGB. Left: entire field of view (white square indicates detail shown in the right panel; scale bar: 100 μm). Right: Detailed view of a subset of labeled neurons. (B) Simultaneous measurement of OGB-fluorescence (ΔF/F, upper trace) and loose-patch recording (lower trace) of a spontaneously firing, putative SPN. The numbers indicate the number of spikes for each detected ΔF/F peak. (C) Relation between the number of extracellularly measured current spikes and normalized average calcium response (linear regression, R^2^ = 0.99, *p* < 0.001, *n* = 10 neurons). The average ΔF/F amplitude for two spikes was normalized to unity due to the lack of single spikes in some neurons. (D) Example ΔF/F traces for a subset of 12 nearby neurons under baseline, PTX (100 μM), and washout. (E) As in D for IEM-1460 (500 μM). (F) Distribution of ΔF/F peaks for PTX (top) and IEM-1460 (bottom). (G) Average ΔF/F peak amplitude for the two conditions (***p < 0.001). (H, I) Same as F and G for the distribution of CCs (*p < 0.05, **p < 0.01, ***p < 0.001). Data for this figure are in S5 Data.

Although the wire arrays used in vivo allowed us to study interactions between striatal neurons, they do not allow for a more detailed analysis of local clusters of neighboring striatal neurons in relation to cortical avalanche dynamics. We therefore recorded intracellular, spontaneous calcium transients in local populations of striatal neurons in these cultures during cortical avalanche activity (12–100 neurons, average: 45.7 ± 1.3, *n* = 11 cultures). Neurons were loaded with the calcium indicator Oregon Green 488 BAPTA-1 (OGB; Fig 5A), and background-corrected calcium transients of spontaneous activity were converted to changes in fluorescence over baseline fluorescence, ΔF/F (see Materials and Methods). Simultaneous loose-patch recordings and calcium imaging (Fig 5B) demonstrated a linear relationship between the number of striatal spikes and corresponding peak ΔF/F amplitudes (Fig 5C), as reported previously [56,57].

Under normal conditions, spontaneous striatal population activity was characterized by irregularly occurring, near-simultaneous episodes in which most neurons participated with largely varying peak amplitudes (Fig 5D, baseline). Amplitude heterogeneity was seen both within episodes and within neurons. Within <30 s of local striatal PTX application (100 μM), peak amplitudes increased (Fig 5F and 5G; *n* = 8, rANOVA, F(2,14) = 36.74, *p* < 0.001) and became highly similar across neurons for each episode (Fig 5D, PTX). This effect was largely reversed after 5 min of drug washout (Fig 5D–5G). Accordingly, the nonselective reduction of fast intrastriatal synaptic inhibition strongly increased the CC between striatal neurons (Fig 5H, top; Fig 5I, rANOVA, F(2,14) = 57.23, *p* < 0.001) in line with our in vivo finding. The unchanged rate of striatal activity episodes during PTX supports intrastriatal location of PTX action (baseline: 0.15 ± 0.02 s^−1^, PTX: 0.14 ± 0.02 s^−1^, washout: 0.15 ± 0.02 s^−1^, *n* = 8, rANOVA, F(2,14) = 0.63, *p* = 0.55; S8 Fig), given that cortical disinhibition would have induced prolonged activity periods at much lower rate in this system [58]. As further control, striatal changes to intrastriatal PTX application did not depend on midbrain inputs, further supporting exclusive intrastriatal PTX action (S9 Fig).

In contrast, when locally applying IEM-1460 to the striatum, average ΔF/F peak amplitudes in the local striatal population did not change (Fig 5E and 5F bottom; Fig 5G, *n* = 11, rANOVA, F(2,20) = 1.77, *p* = 0.195), and the average CC between neurons did not increase (Fig 5H, bottom; Fig 5I, rANOVA, F(2,20) = 6.88, *p* = 0.005; CC_baseline_ > CC_IEM-1460_ > CC_washout_). These in vitro results demonstrate that, in the presence of cortical avalanches, striatal neurons show low CCs that depend on local GABA_A_-mediated inhibition and were not abolished after reduction of striatal IN-mediated inhibition. It confirms our initial results in vivo that nonselective intrastriatal disinhibition increases striatal synchrony, whereas a decorrelated striatal resting state is maintained after disruption of IN-mediated inhibition.

### Transition between Striatal States of Pairwise Correlations in Response to IEM-1460

**Fig 6:**
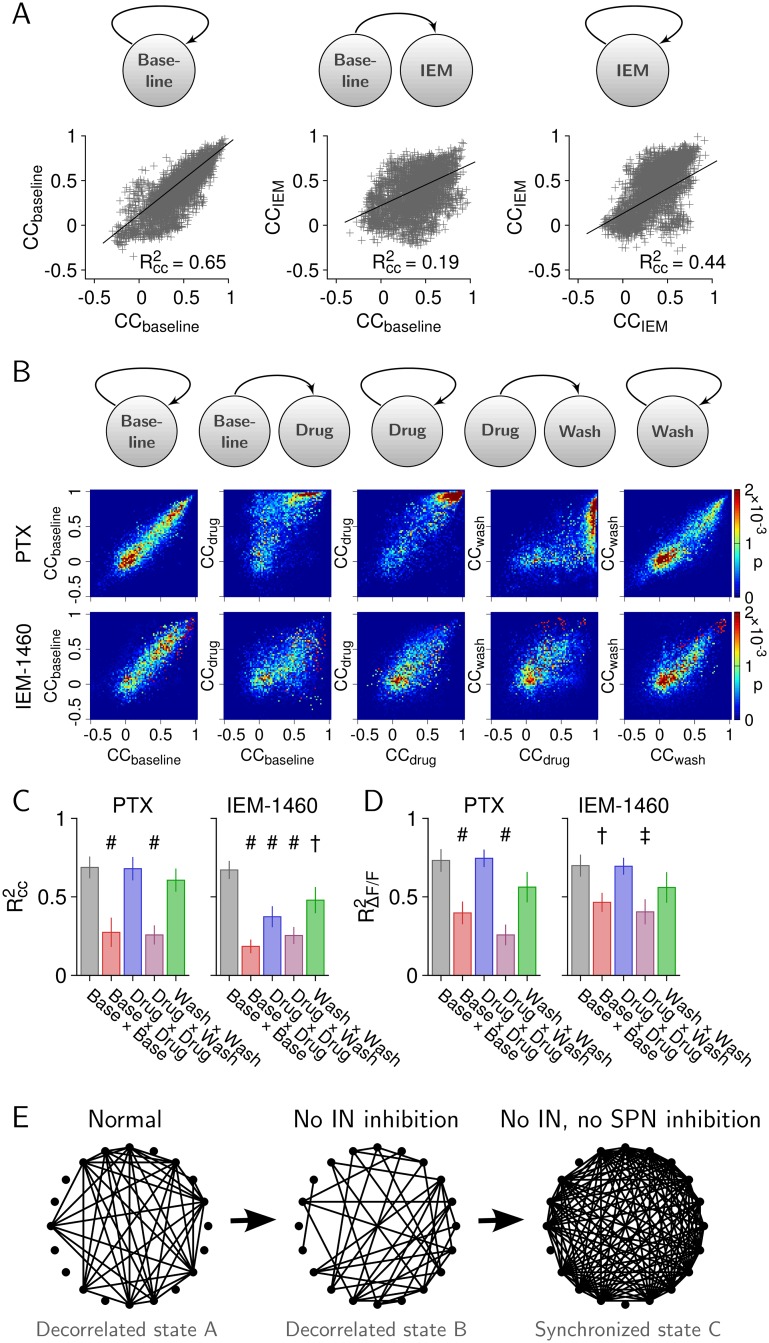
IEM-1460 changes the functional connectivity between striatal neurons without changing the decorrelated state. (A) A set of CCs (left, baseline) breaks up (middle) and stabilizes as a new set upon application of IEM-1460 (right). Left: First half of baseline versus second half of baseline. Middle: Second half of baseline versus first half of IEM-1460. Right: First half of IEM-1460 versus second half IEM-1460. Changes are quantified using R^2^ for CCs obtained during consecutive time segments (~2 min each) for one culture (*n* = 3,570 pairs). (B) Probability density maps of CCs for PTX (*n* = 8 cultures) and IEM-1460 (*n* = 11 cultures). (C) Average $R_{CC}^{2}$ values for PTX (left) and IEM-1460 (right). (D) Same as C for changes in ΔF/F amplitude in individual neurons (^#^*p* < 0.001, ^†^*p* < 0.01, ^‡^*p* < 0.05; comparison to baseline×baseline, Bonferroni-corrected). (E) Illustration of the functional networks under normal condition and during striatal disinhibition (dots indicate 20 randomly selected SPNs, lines indicate CCs above 0.7). Blockade of IN-mediated inhibition changes the functional connectivity without abolishing the decorrelated state (state A→B). Blockade of IN- and SPN-mediated inhibition results in a strongly synchronized state C. Data for this figure are in S6 Data.

The previous analysis provides a picture of average changes but, in general, does not capture individual alterations in ΔF/F amplitude of single neurons or pairwise correlations (i.e., CCs) between neurons [59]. That is, different constellations of amplitudes or correlations could result in the same average. Indeed, the inability of IEM-1460 to change the average CC in the striatum was contrasted by its ability to significantly change individual CCs between neurons, that is, to reorganize the functional connectivity of the striatum while maintaining a low-correlation resting state. This is illustrated in more detail in Fig 6A, in which CC was quantified for consecutive segments of ΔF/F of each ~2-min duration. CC values from consecutive segments were plotted, and the coefficient of determination, $R_{CC}^{2}$, was calculated using linear regression. A value of $R_{CC}^{2}$ close to one indicates little change of individual CCs between segments, whereas $R_{CC}^{2}$ towards zero indicates a strong change. The value of $R_{CC}^{2}=\;0.65$ in Fig 6A provides a reference value for the expected change of CCs within a few minutes for a single culture. In this example, the comparison baseline versus IEM-1460 yielded a reduced value of $R_{CC}^{2}=\;0.19$ (Fig 6A, middle), demonstrating that individual CCs changed upon local application of IEM-1460, as can be seen in the corresponding scatterplots. Fig 6B shows density plots of CCs for all consecutive segments and cultures (PTX: *n* = 8; IEM-1460: *n* = 11). The corresponding $R_{CC}^{2}$ values are summarized in Fig 6C, demonstrating that, similar to PTX, IEM-1460 led to a highly significant change in CCs (rANOVA; PTX: F(4,28) = 16.21, *p* < 0.001; IEM-1460: F(4,40) = 15.72, *p* < 0.001). The analysis of the change in individual ΔF/F peak amplitude averages revealed a similar picture. That is, although IEM-1460 did not lead to changes in the grand average ΔF/F (Fig 5G), it changed the ΔF/F responses in individual striatal neurons significantly (Fig 6D, rANOVA, PTX: F(4,28) = 21.79, *p* < 0.001; IEM-1460: F(4,40) = 5.38, *p* = 0.0015). That changes in individual CCs under IEM-1460 as quantified by R^2^ were of similar magnitude compared to PTX (Fig 6C and 6D) further suggests that the lack of synchronization under IEM-1460 cannot be explained by insufficient blockade of IN inhibition.

In summary, these results strongly suggest that, under normal conditions, the low-correlation state among striatal neurons requires local GABA_A_-mediated inhibition and that reduction of spontaneous IN firing changes the pairwise correlation state while maintaining a low average correlation (Fig 6E).

## Discussion

Movement disorders, in which the basal ganglia play a pivotal role, remain a significant public health burden [25]. Although our study confirms that suppression of involuntary movements requires intact striatal inhibition [25,38,41,48], here, we demonstrate two vastly different mechanisms of the emergence of involuntary movements when manipulating striatal inhibition. The particular dynamics that gave rise to involuntary movements involved cortico-basal ganglia loops, which, due to their recurrent feedback nature, made the analysis of the population dynamics particularly challenging from a systems’ point of view. A combined in vivo and in vitro approach using microelectrode array recordings and cellular resolution population imaging enabled us to study striatal population dynamics under closed- and open-loop conditions. Through this combination of techniques, we identified a low-correlation resting state of the striatum stabilized by striatal INs from which, after disruption of IN inhibition, involuntary movements can emerge at high rate involving cortico-basal ganglia loops. These pathological dynamics might be of particular clinical relevance for humans suffering from Tourette syndrome who reveal a reduction in striatal IN number [39,40]. This scenario was distinguished from a nonselective disinhibition in the striatum, which significantly increased striatal synchrony and gave rise to dyskinetic movements at much lower rate, a condition that might be encountered in early stages of Huntington’s disease when striatal neurons degenerate [22].

### Differential Effects of PTX and IEM-1460 on Cortico-Basal Ganglia Dynamics and Behavior

In the current study, we used two pharmacological agents that affect striatal inhibition. The first, PTX, blocks GABA_A_ receptors expressed in SPNs and INs. In the past, GABA_A_-antagonists have been extensively used to study the effect of striatal disinhibition on neuronal firing and motor behavior [41–45]. The second drug, IEM-1460, influences striatal inhibition indirectly by blocking AMPA-mediated inputs to striatal inhibitory INs, thus leading to the disinhibition of SPNs by reducing IN-to-SPN activity. Although PTX also affected the latter connection (i.e., IN-to-SPN synaptic transmission), we observed in our experiments distinct activity and behavioral phenotypes. In vivo, both substances increased striatal synchrony and induced involuntary movements. However, local striatal PTX increased striatal synchrony even in the absence of a closed corticostriatal loop (via globus pallidus/substantia nigra/thalamus), leading to highly synchronized striatal events in our in vivo and in vitro preparation. In contrast, local striatal IEM-1460 showed increased synchrony only in vivo, most likely due to increased synchronous input from cortex (and thalamus). That is, local striatal IEM-1460 application deviated cortical activity away from avalanches into a highly synchronized state not observed under PTX. In addition, PTX and IEM-1460 showed neuronal bursts and involuntary movements at very different frequencies. That even low doses of IEM-1460 (0.5 mM versus 5 mM) induced striatal bursts at high rate further suggests that, indeed, PTX and IEM-1460 influence cortico-basal ganglia loop activity in very different ways. Thus, although both substances induced involuntary movements, our findings suggest distinct mechanisms underlying the emergence of these movements. We also note that the relatively high frequency of involuntary movement components under IEM-1460 could suggest tremor-like spontaneous movements. However, due to their variability (S2 Movie) and intermittency (S2 Fig), these movements do not resemble a continuous tremor. These mechanisms and behavioral phenotypes need to be further explored in future studies to improve the understanding of normal and pathological conditions in the basal ganglia.

### A Low-Correlation Striatal Resting State That Supports Cortical Avalanche Dynamics In Vivo

Our combined in vitro and in vivo findings identified a low-correlation resting state of the striatum that is maintained in the presence of cortical neuronal avalanches and depends on intrastriatal inhibition. Avalanche dynamics in cortex are characterized by long-range spatial and temporal correlations and are described by a power law in burst size distribution with exponent close to –1.5 [4]. The low-correlation striatal resting state dynamics qualitatively differed from cortical neuronal avalanche activity as measured by a more negative power-law exponent in vitro, indicating a spatially more confined activation of striatal neuronal populations compared to cortex. Our results further show a differential participation of two major striatal microcircuit components in maintaining and regulating ongoing striatal and cortical avalanche activity through cortico-basal ganglia-thalamic loops during resting. This finding relied on a precise quantification of the cortical resting state, that is, the measurement of cortical avalanches and the quantification of deviations from avalanche dynamics. Avalanche dynamics are robustly identified using the LFP at the mesoscopic level, although recent advances with single-cell resolution have been obtained for cortex [7]. We confirmed that cortical LFPs are related to local neuronal activity and that they organize as neuronal avalanches both in vivo and in vitro.

The low rate of neuronal population bursts and corresponding involuntary movements induced by nonspecific reduction of intrastriatal inhibition is due to a refractoriness of cortex, with an absolute refractory period >300 ms [45]. Disfacilitation of inhibitory striatal INs using IEM-1460, which left the feedback inhibition between SPNs intact, quickly abolished cortical neuronal avalanche dynamics and induced corticostriatal population bursts, often less than 300 ms apart [60]. This suggests that the striatum might require lateral inhibition between SPNs to efficiently entrain cortical activity at a subsecond time scale. This finding leads us to propose that it is not the change in average correlation or activity in striatal output but rather the specific functional connectivity of the striatum, supported by lateral SPN inhibition, that influences ongoing cortical avalanche dynamics, presumably via substantia nigra/globus pallidus and thalamus by promoting certain avalanche patterns in cortex.

### Pharmacological Approach Using IEM-1460 to Reduce Striatal IN Firing

The striatum receives excitatory and inhibitory input from various sources that are part of intricate feedback loops, such as cortex [61,62], thalamus [63], globus pallidus [64], and substantia nigra [65]. Using an open-loop in vitro system that exhibits the same resting state dynamics as in vivo, i.e., cortical neuronal avalanches, allowed us to isolate those aspects of striatal dynamics and corresponding microcircuits that underlie the observed dynamical changes in vivo. We confirmed in vitro that nonselective reduction of intrastriatal inhibition using PTX synchronizes striatal action potential firing, in line with the striatal changes at the multi-unit and LFP levels in vivo. Importantly, we could demonstrate in vitro that avalanche-induced striatal firing remains decorrelated when reducing IN firing with IEM-1460, in line with a low increase in relative striatal synchrony observed in vivo under those conditions (Fig 2E). Taking advantage of monitoring local clusters of striatal neurons at high spatial resolution using intracellular calcium imaging, our in vitro approach allowed us to dissect the apparent discrepancy between the strong effect of IN manipulation in the absence of major changes in striatal synchrony. We could demonstrate in vitro that IEM-1460 strongly affected which neurons were (co-) active with little or no influence on the average CC between striatal neurons and average single neuron response amplitudes, respectively. We believe that this finding introduces the specific functional connectivity maintained dynamically by local striatal inhibition as a major factor in the regulation of activity in cortico-basal ganglia loops.

We note that changes in single neuron activity under IEM-1460, quantified by the coefficient of determination, were comparable to PTX (Fig 6C and 6D). In addition, local IEM-1460 injections in vivo induced strong changes in striatal neuron activity at high as well as ten times lower concentrations, with corresponding strong changes in cortical avalanche dynamics. This suggests that even small reductions in IN inhibition induce strong dynamical changes in cortico-basal ganglia loops.

In addition to the reduction of AMPA-mediated currents for receptors lacking the glutamate receptor 2 (GluR2) subunit, IEM-1460 has been shown to reduce NMDA receptor currents at high concentrations, thereby possibly influencing SPN input. However, the effectiveness of blocking NMDA currents is two orders of magnitude smaller compared to the effect on AMPA currents [66]. Importantly, such NMDA receptor blockade would have been expected to decrease SPN neuron firing. However, our in vitro results showed no change in average response amplitude in SPNs, and a subset of SPNs even increased their responses to IEM-1460 application (see also [38]). IEM-1460 has been shown to target AMPA receptors in cholinergic INs, and cholinergic neurons via inhibitory neuropeptide Y-positive neurogliaform neurons can influence SPN firing [67–69]. However, the absence of correlations between cholinergic INs and SPNs in nonhuman primate recordings [70] suggests that this pathway might not dominate striatal resting activity in vivo or in general during cortical neuronal avalanches. Accordingly, even after blockade of cholinergic transmission, selective reduction of IN firing using IEM-1460 can induce hyperkinesia [38].

Our pharmacological approach in vitro allowed for the manipulation of intrastriatal circuits in the absence of cortico-basal ganglia feedback loops and inhibitory inputs originating from globus pallidus or midbrain. However, our approach is not able to exclude other possible sources that might contribute to the observed decorrelation effect, such as inputs from diverse striatal IN classes or from a newly described corticostriatal inhibitory pathway [71]. While cell–specific manipulations can be achieved in striatal SPNs and INs using optogenetic techniques in transgenic mice [67,72], changing SPN firing to precisely test whether inhibition between SPN maintains an intrastriatal low-correlation resting state currently faces two caveats. First, manipulation of SPN firing does not allow differentiating between intrastriatal (i.e., SPN-to-SPN) and loop (i.e., striatopallidal/-nigral) connectivity. Second, SPN firing was used as a readout of the network state to calculate response amplitudes and CCs. Dissecting the functional role of feedback inhibition between SPNs would require an opto- or pharmacogenetic approach that directly manipulates SPN-to-SPN synapses, for which techniques are currently being developed [73,74].

In our in vivo experiments, MUA did not allow us to differentiate striatal cell types involved, and even single-unit analysis in the striatum is limited in mapping waveforms to identifiable cell types [75,76]. Although our multi-unit and LFP analyses identified the differential effect of PTX and IEM-1460 on cortico-basal ganglia loops, we were unable to demonstrate the corresponding specific changes in striatal IN firing. Naïvely, one might assume that the net effect of IEM-1460 in vivo reduces IN firing; however, as shown by the dramatic changes in loop activity, loop reverberation does not permit interpretations of changes in IN activity based on direct drug action alone. Therefore, we extended our in vivo approach to in vitro, in which we recorded up to a hundred striatal neurons at single-cell resolution in an open-loop configuration to further quantify the network changes in the striatum. These in vitro results showed reduced IN firing during spontaneous avalanche dynamics under IEM-1460 as well as a decorrelated striatal state. The open-loop findings in vitro are in line with a reduced glutamatergic drive of INs and the limited role of INs in decorrelating striatal activity.

### Potential Cellular Mechanisms That Maintain a Low-Correlation Striatal Resting State and Prevent the Emergence of Involuntary Movements

The average low correlation in the striatum was maintained when manipulating INs, supporting the view that lateral (i.e., feedback) inhibition between SPNs [28–30] might be responsible for the low-correlated striatal resting state, in line with prediction from theory and simulations [31,32]. That feedback inhibition can affect the population of striatal neurons was indeed shown in acute slices through antidromic electrical activation [77] (with potential contributions from pallidal-striatal projections [64]). The increase in striatal synchrony upon nonselective reduction of intrastriatal inhibition using PTX is also in line with another study [78], in which acute slices were activated by electrical cortical stimulation or NMDA receptor activation.

Computational studies suggest that networks of inhibitory neurons with realistic connectivity regimes for lateral inhibition reduce the level of activity, increase the contrast of responses, i.e., decorrelate striatal input [79,80], and can cause transitioning between striatal cell assemblies [32,81]. Although lateral inhibition is not the only mechanism by which neuronal network activity can be decorrelated [82], it can greatly enhance pattern decorrelation, as recently shown in a computational network model of neurons with threshold nonlinearities [33]. Thus, threshold nonlinearities [83] and corticostriatal connectivity [62] are likely to contribute to the observed decorrelation in striatal activities.

The above models have in common that they require the collective inhibitory influence of cell groups. We propose that the large number of SPNs and wide distribution of measured synaptic strengths [35,36,84] provide the basis for lateral inhibition to affect striatal output and, consequently, future cortical activity [85]. Striatal inhibitory INs, on the other hand, might influence the functional connectivity of SPNs, thus promoting changes between different states of low correlation in the striatum that might encode specific motor programs. This idea is supported by a recent study [86], which found that striatal fast-spiking INs increase their firing particularly during the choice execution period in a choice task.

In the context of our study, we propose that a change in IN activity promotes switching between low-correlation states in the striatum, which entrain cortico-basal ganglia loops supported by lateral inhibition between striatal projection neurons. In summary, our results uncover different dynamical influences of two major intrinsic striatal microcircuits in regulating cortico-basal ganglia resting activity important for the suppression of involuntary movements in normal behavior.

## Materials and Methods

All animal procedures were in accordance with National Institutes of Health guidelines. Animal procedures (protocol numbers LSN-01 and LSN-12) were approved by the National Institute of Mental Health Animal Care and Use Committee.

### In Vivo Recordings

Male Sprague-Dawley rats (5–8 wk old) were used for behavioral assessment and/or chronic recording of LFPs and MUA in the cortex or striatum. To study the influence of the striatal inhibitory mechanisms, two different substances were microinjected into the dorsal striatum (AP: 0.9–1.5 mm, ML: 2.2–2.6 mm, 4.2–5.5 mm from cortical surface) through a chronic cannula (26 gauge, 1–2 mm projection; Plastics One, Roanoke, VA, United States): (1) PTX (Sigma-Aldrich), a GABA_A_-receptor antagonist, and (2) IEM-1460 (Tocris Bioscience), an antagonist of GluR2-lacking AMPA receptors selectively expressed in striatal INs [46,47,87]. Implantation of the cannula guide and the recording array was done under isoflurane anesthesia (1.5%–4%, 100% oxygen) and presence of the analgesic ketoprofen (5 mg/kg, subcutaneous). All cannula guides, cannulas, and recording arrays were sterilized. During the implantation surgery, care was taken to avoid blood vessels. To prevent unnecessary brain injury, the dura was carefully ruptured and the cannula guide and/or recording array was slowly lowered at a rate of ~150–200 μm per min. Ketoprofen was given for up to 2 d post surgery, and animals were allowed to recover for 2–5 d before recordings. Spontaneous activity for LFP and MUA analyses (see below) was recorded up to 2 wk post surgery from superficial layers of the primary motor cortex and/or the somatosensory forelimb region (AP: 0.5–2.2 mm, ML: 3.2–3.5 mm, and 0.2–1.1 mm from cortical surface) using 8×4-MEAs (8 shanks with 4 electrodes each, plus additional reference electrode implanted along the anterior-posterior axis; 28–32 working electrodes; 200 μm inter-electrode spacing; 23 μm electrode diameter; Neuronexus, Ann Arbor, MI, US), or the dorsolateral striatum (AP: 0.7–2.1 mm, ML: 3.2–3.5 mm, 3.2–3.5 mm from cortical surface) using 16-channel MWAs (8×2 electrodes plus additional reference wire implanted along the anterior-posterior axis; 14–16 working electrodes; 150 μm inter-wire distance; 0.6–0.9 MΩ impedance; Microprobes, Gaithersburg, MD, US). The ground wire was connected to a scull screw located ~1 mm posterior to lambda. Data were recorded for at least 30–60 min at 30 kHz using a Cerebus data acquisition system (Blackrock Microsystems). After baseline recordings, 0.8–1.5 μl sterile drug solution (PTX, 1 mM; IEM-1460, 5 mM) was injected at a rate of 0.3 μl/min for 3–5 min. The internal cannula was left in place for 1–2 min post injection, after which recordings were performed. Animals were allowed to recover for 1 d before the next recording session. In *n* = 3 rats, we tested a ten times lower IEM-1460 concentration (0.5 mM) and found that two out of three rats showed involuntary movements that were of similar nature as under higher IEM-1460 concentration, that is, intermittent movements at high rate in the contralateral front paw.

Animal behavior was video-recorded with a Logitech c920 camera (10–30 frames per s, fps) for behavior-only recordings, or simultaneously with LFP and MUA using a triggered CMOS camera (40 fps; Thorlabs). Involuntary movements were analyzed using custom scripts in Matlab (Mathworks, MA, US). We defined a “movement” signal as 1 minus the frame-to-frame correlation for a region of interest (i.e., contralateral front paw or neck), and involuntary movements were extracted by applying a threshold of 2–3 SDs (Fig 1C and 1D). Only periods during which animals were resting (i.e., no locomotion, cage exploration, or grooming) were included in the video analysis. After recordings were finished, brains were dissected and the locations of cannula and electrode placements were confirmed in a subset of animals.

In total, data from 17 rats were analyzed in this study. All but one rat were chronically implanted with a cannula guide for local drug infusion in the striatum. A subset of rats was implanted with an MWA in the striatum (*n* = 8 rats) or an MEA in the superficial layers of cortex (*n* = 5 rats). A list of all rats and the recordings and observations is given in S1 Table.

### Organotypic Culture Preparation

Coronal slices from rat cortex (350 μm thick, postnatal days 0–2; Sprague Dawley), striatum (500 μm thick), and midbrain (substantia nigra pars compacta; 500 μm thick) were cut on a vibratome (VT1000 S, Leica, Wetzlar, Germany) in ice-cold, sterile Gey’s balanced salt solution (0.4% D-glucose) and cultured on poly-D-lysine coated and plasma-/thrombin-treated carriers to allow proper tissue adhesion [52]. After tissue adhesion to the carrier, standard culture medium was added (600 μl of 50% basal medium, 25% HBSS, 25% horse serum, 0.5% glucose, and 0.5% of 200 mM L-glutamine; Sigma-Aldrich) and changed every 3–4 DIV. At 1, 8, and 20 DIV, 10 μl mitosis inhibitor (0.3 mM uridine, 0.3 mM ARA-C cytosine-β-D-arabinofuranoside, and 0.3 mM 5-fluoro-2′-deoxyuridine) was added for 24 h to prevent excess glia cell formation. Cultures were incubated at 35.5 ± 0.5°C. Carriers were either coverslips for calcium imaging and patch recording experiments or 60-channel, planar MEAs for the recording of LFPs and MUA (see below). Cultures on coverslips were incubated in a roller tube incubator at 0.6 rotations/min, and MEA cultures were incubated on a rocking storage tray at ±75°, 0.25 cycles/min (±25°, 0.6 cycles/min during the recording sessions except for developmental data, Fig 3C).

### In Vitro Multielectrode Array Recordings

Planar titanium nitride MEAs with 60 channels (59 recording electrodes plus one reference electrode; 200 μm inter-electrode distance, 30 μm electrode diameter) were obtained from Multichannel Systems (Reutlingen, Germany). For the developmental recordings, a standard 8×8 layout was used. For all other MEA recordings, a custom layout with two sub-arrays for cortex (8×4, 31 electrodes) and striatum (6×5, 28 electrodes) was used. Both sub-arrays were separated by 1,200 μm (Fig 3A). Data were recorded at 25 kHz for MUA or 1 kHz for LFP using an MEA1060 amplifier and the MC Rack software (Multichannel Systems). Spontaneous activity for the developmental data (20 min) and the experiments with PTX bath application (4 μM, 60 min) was recorded in culture medium under sterile conditions. Washout recordings were done 24–48 h after the culture medium was replaced with conditioned medium collected 3–4 d before the experiment. All recordings were performed at 35 ± 0.5°C after 2 wk in vitro if not stated otherwise.

### Calcium Imaging

Calcium imaging was performed on coverslip cultures loaded with 50 μM OGB (Life Technologies, NY, US) dissolved in 10 μl pluronic F-127 (20% in DMSO; Life Technologies, NY, US) and 790 μl freshly prepared artificial cerebrospinal fluid (ACSF) [88]. ACSF was bubbled with 95% O_2_ and 5% CO_2_ and contained (in mM): 124 NaCl, 3.5 KCl, 10 D-(+)-glucose, 26.2 NaHCO_3_, 0.3 NaH_2_PO_4_, 1.2 CaCl_2_, and 1 MgSO_4_. Cultures were incubated for 60–90 min in a roller tube incubator and perfused with ACSF (flow rate ~100 ml/h) for 20–30 min before imaging. After baseline recordings (5 min), PTX (100 μM) or IEM-1460 (500 μM) was ejected locally in the striatum at a rate of 12 μl/min for 5 min using glass pipettes with a tip diameter of ~80–100 μm, while intracellular calcium was simultaneously imaged. Washout conditions were recorded 10–20 min after the drug application ended. Drug spillover to the cortex was prevented by using a two-compartment chamber in which a glass coverslip separated the bath between the cortical and striatal tissue. The glass coverslip was positioned ~300 μm above the tissue and sealed with agar pieces around the recording chamber. ACSF and drug flow was directed away from cortex. This approach was highly efficient in avoiding any drug spillover to cortex, as shown in S8 Fig. All recordings were performed between 13–28 DIV.

Image sequences (12 bit, 2×2 binning, 320×240 pixels) were acquired with a Peltier-cooled CCD camera (Imago from TILL Photonics, Gräfelfing, Germany) on an inverted microscope (Olympus IX70) with a 20× water-immersion objective (numerical aperture 0.7). Excitation wavelength was set to 492 nm using a monochromator (Polychrome II, TILL Photonics). Excitation, dichroic, and emission filters from Omega Optical (Brattleboro, VT, US) were XF1087 (445–495 nm band-pass), XF2077 (reflection <500 nm), and XF3105 (508–583 nm band-pass), respectively. Image sequences of up to 320 s (7,000 frames) were obtained at a rate of 21.7 frames/s (cycle time 46 ms, exposure 28 ms) using the TILLvisION 4.0 software (TILL Photonics). Image sequences were converted into TIF file format after acquisition and analyzed in Matlab using custom scripts.

Regions of interest (ROIs) were manually selected by identifying typical cell bodies (Fig 5A), and background subtraction was performed by automatically subtracting the fluorescence signal from a dark background region within the area of two cell body diameters. All fluorescence values are expressed as relative change in fluorescence from baseline, denoted by Δ*F/F*, and measured as percentage. Formally, Δ*F/F* is defined as the percentage change in fluorescence over baseline, that is, Δ*F/F* = *100* (*F*_*ROI*_−*F*_0_)/*F*_0_, where *F*_*ROI*_ and *F*_0_ denote the background-corrected fluorescence intensities in the ROI and the baseline, respectively. For the spike-triggered detection of fluorescence changes (Fig 5B and 5C), the baseline was calculated as the average fluorescence 50 ms before the spike. For all other analyses, the baseline was calculated from a 30-s sliding window as the average of the 50% smallest values (i.e., excluding transients that correspond to neuronal activity). To allow for a more robust detection of calcium transients, successive increases in fluorescence (Δ*F/F* > 0) were summated, and the threshold detection was performed on this summated signal. The percentage of spuriously detected Δ*F/F*-peaks was lower than 0.5% (*n* = 8 neurons).

For the estimation of the rate of up-state events in striatal neurons, we used the summed widefield (bulk) fluorescence signal within the field of imaging. Because up-states among striatal neurons are correlated [51,89] and driven by cortical input, this approach gave a good approximation of the input rate arriving from cortex.

### Patch-Clamp Recordings

Patch pipettes were pulled from borosilicate glass using a P-97 micropipette puller (Sutter Instrument, CA, US), and had a resistance of 5–10 MΩ. For all recordings, pipette resistance and capacitance were compensated for. Loose-patch and cell-attached recordings were performed in voltage-clamp mode using patch pipettes that were filled with regular ACSF. Whole-cell patch-clamp recordings were done in current-clamp mode with an intracellular solution containing (in mM) 132 K-gluconate, 6 KCl, 8 NaCl, 10 HEPES, 2 Mg-ATP, and 0.39 Na-GTP, or voltage-clamp mode with an intracellular solution containing (in mM) 132 CsMeSO_3_, 1 CsCl, 10 HEPES, 2 Mg-ATP, 0.39 Na-GTP, and 5 QX-314. The intracellular solution was kept on ice during the experiment. For the local application of PTX (100 μM) or IEM-1460 (500 μM), a second patch pipette was placed in close vicinity (<60 μm; Fig 4A) of the patched soma and drugs were ejected at 50–55 mmHg. As expected, all recorded neurons (5/5) responded to ejection of DNQX (50 μM, Fig 4E). Significance was calculated by comparing 100 s baseline plus 100 s washout with 80 s of drug data using Student’s *t* test.

### Immunohistochemistry and Confocal Imaging

A subset of cultures was used for post-hoc immunostaining of TH. Cultures were rinsed in phosphate buffered saline (PBS), fixed in 4% paraformaldehyde for 40–60 min, and incubated for 2 h at room temperature in blocking solution (10% normal goat serum and 0.5% Triton X-100 in PBS). For all subsequent steps, a carrier solution consisting of 1% normal goat serum and 0.3% Triton X-100 in PBS was used. Cultures were incubated for ~12 h at 4°C in a TH-antibody solution (1:1000, antimouse, Immunostar, WI, US), washed three times for 10 min each, incubated 1–2 h at room temperature in secondary antibody solution (1:1000, Alexa 555 anti-mouse, Invitrogen, NY, US), and washed again three times for 10 min each at room temperature. Before the confocal imaging, cultures were rinsed in PBS and mounted on coverslips using a fluorescence-preserving mounting medium (Vector Laboratories, CA, US).

Confocal images were obtained with a Zeiss LSM 510 using a 63× oil immersion objective (numerical aperture 1.4, 0.6 μm optical thickness). For the cell counting of TH-positive neurons in the substantia nigra pars compacta, images were obtained with a high-speed scanning confocal microscope (Leica TCS SP5 II, 10× objective) with tile scan function. Cell counts were obtained from maximum z-stack projections (42 μm thick, 6 μm optical thickness). For TH-positive neurons that were organized in dense clusters, only well-distinguishable somata or somata with distinct (dark) nucleus were counted (Fig 3B, right panel), thus likely underestimating the actual number of dopaminergic neurons.

### LFP and MUA Analyses

For in vitro recordings, MUA was detected by band-pass filtering at 300–4000 Hz and subsequent thresholding (–5 SD of each trace). LFPs were band-pass filtered at 1–100 Hz for the developmental data, which contained dominant frequency components above 50 Hz, and 1–50 Hz for all other analyses. nLFP deflections were detected by finding the minimum value of the LFP signal that crossed a given threshold z (measured in SDs). Previous studies showed that cortical nLFPs are associated with increased activity and synchrony in local firing [8,10,49]. That cortical nLFP are correlated with cortical multi-unit firing was confirmed in this study (S5 Fig). We furthermore found this relationship in the striatum in vitro (Fig 3D) despite small amplitudes of striatal nLFPs. The SD was determined for each channel individually and estimated from 2–3 s of baseline activity (z = –4.5). The threshold value z was varied to confirm the robustness of the reported power-law exponents (see also [10,50]). For the power spectral analysis of the developmental data, ±500 ms around nLFP threshold crossings were analyzed. The power spectrum was calculated by using the fast Fourier transform with a Hann window function. Averages for individual cultures were calculated across all channels and subsequently normalized (integral over the entire frequency range normalized to unity) before calculating the average over all cultures. All in vitro data were analyzed using the phase-neutral filter implementation *filtfilt* in Matlab and the Neuroshare library (http://neuroshare.sourceforge.net) for data import.

For in vivo recordings, presumable multi-unit spikes were extracted from the high-pass filtered signal (>250 Hz) by applying a threshold at –6 times the root mean square of the signal using the Cerebus Central software (Blackrock Microsystems). Because movements could cause artifacts in the high-pass filtered signal, thresholded waveforms were subsequently offline-sorted using the Offline Spike Sorter (Plexon Inc., Dallas, TX, US). Only electrodes were used for analysis for which MUA could be isolated from movement artifacts based on the typical biphasic waveform of multi-unit spikes (Fig 1G, inset). Calculation of LFPs was performed as described for in vitro using the entire signal for each electrode for the estimation of SD (z = –2.5). For the avalanche analysis, z was varied to confirm the robustness of the estimated power-law exponents (see above). In the striatum, MUA was associated with pLFP deflections (S4 Fig; Fig 1G, left). We therefore extracted pLFP deflections (z = 2.5–3) from in vivo striatal recordings.

### CC Analyses

CCs were calculated from binned time series (rasters) of p/nLFPs, multi-unit spikes, or from continuous Δ*F/F* traces. Values for p/nLFP and MUA rasters were discrete and corresponded to the number of p/nLFP or spike events per bin, respectively. The raw CC between two time series, *x*_*t*_ and *y*_*t*_, was defined as

$$CC\left(\tau \right)=\;\frac{E\left[\left(x_{t}-\mu_{x}\right)\left(y_{t+\tau }-\mu_{y}\right)\right]}{\sigma_{x}\sigma_{y}}$$

where *E*[·] denotes the expected value operator, τ the time lag, and μ and σ denote mean and SD, respectively. CC for n/pLFP or MUA rasters were shuffle-corrected by subtracting *CC*_*shuffle*_ (average of ten repetitions) from *CC*. The calculation of CC for calcium imaging data was performed on the Δ*F/F* traces. The average CC was reported as the average value across all electrode or neuronal pairs for time lag τ = 0 if not stated otherwise. In Fig 6, individual CCs were analyzed.

### Detection of Spatiotemporal nLFP Clusters and Avalanche Analysis

Rasters of nLFP events that crossed a predefined threshold, z, were created by binning the nLFP times with bin size Δt = 2–4 ms [4,11,50]. Previous studies showed that cortical nLFPs can be used as a readout of cortical synchronized population activity (see also S5 Fig) [8,10,49] to measure the propagation of spatiotemporal activity clusters. Due to the predominantly local propagation of activity [11], compact 8×4 MEAs (Fig 2A in vivo, Fig 3A in vitro) were used as described above. From the recorded nLFP rasters, spatiotemporal clusters were extracted by finding cascades of nLFP events that were separated by at least one bin width (Fig 2F). The size of a cluster was defined as the number of nLFPs within the cluster (“discrete,” Figs 2G and 3H, left). Alternatively, cluster sizes can be defined as the sum of absolute nLFP amplitudes (“continuous,” measured in μV; Fig 3H, right), resulting in a continuous distribution [4]. Neuronal avalanches are defined by a distribution of cluster sizes that follows a power law with exponent –1.5 [4] up to the number of electrodes in the recording array. Importantly, the power law is invariant to the number of electrodes used in the recording array up to the so-called “cut-off,” which is given by the number of electrodes in the recording array. This property allows for a robust estimation of the power law exponent [11,50], as described below.

### Statistical Analyses

Power-law exponents were estimated using a maximum-likelihood approach [50,90]:

$$\hat{\alpha }=\underset{\alpha }{\text{arg max}}\;l(\alpha |s)$$

where

$$l\left(\alpha |s\right)=\;\overset{n}{\underset{i=1}{\sum }}\text{ln }p_{\alpha }\left(s_{i}\right)$$

denotes the log-likelihood of observing the vector of given cluster sizes ***s*** = (*s*_1_,…,*s*_*n*_) assuming a power law with exponent α, that is,

$$p_{\alpha }\left(s\right)=\frac{s^{\alpha }}{\sum_{x=1}^{N}x^{\alpha }}$$

In cortical networks, the cut-off is typically at the system size, *N*, which is given by the number of electrodes in the cortical array (see [4,11,50]). Thus, cortical event size distributions were fitted on the range from one to the number of electrodes in the cortical array. Correspondingly, exponents for striatal distributions are reported for a model that ranged from one to the number of electrodes in the striatal array.

For the comparison of power law versus exponential distribution (the expected distribution for independent neuronal activity), we used the LLR test [50,90]:

LLR(s) =l(α|s) −l(λ|s)

where *l*(α|***s***) denotes the log-likelihood for a power law with exponent α, and *l*(λ|***s***) the log-likelihood for an exponential distribution with parameter λ

$$p_{\lambda }\left(s\right)=\;\frac{e^{-\lambda s}}{\sum_{x=1}^{N}e^{-\lambda x}}$$

For the comparison of distributions to a power law with exponent –1.5, or across different experimental conditions, we used the KS statistic [50]

$$D_{KS}=\underset{x}{\text{max}}\left|P_{data}\left(x\right)-P_{compare}\left(x\right)\right|$$

where *P*_*data*_ denotes the cumulative distribution of the data and *P*_*compare*_ the cumulative distribution of the reference power-law model [i.e., $P_{compare}\left(x\right)=\sum_{s=1}^{x}p_{\alpha }\left(s\right)$] or data from a different experimental condition.

For paired comparisons of two or more means, we used the paired Student’s *t* test and repeated-measures ANOVA with Bonferroni correction, respectively. Values are expressed as mean±standard error of the mean if not stated otherwise.

## Supporting Information

S1 Fig Reconstruction of cannula and electrode array locations (*n* = 8 rats). (TIF)

S2 Fig Involuntary movements and LFP burst activity show tighter coupling under PTX compared to IEM-1460. (A) Example of simultaneously recorded LFP (average) and involuntary movements in the contralateral front paw after local striatal injection of PTX (1 mM). (B) The same as in A for a different rat under IEM-1460 (5 mM), showing the intermittency of involuntary movements in the presence of continuous oscillatory striatal LFP activity. Note the different time scales in A and B. For the calculation of the “movement” signal, see Materials and Methods.
(TIF)

S3 Fig IEM-1460 induces fast LFP fluctuations at ~0.2 s (see peak in the autocorrelation functions, arrows) under regular (5 mM) and ten times lower dose (0.5 mM). Left column: example LFP traces. Right column: average autocorrelation functions for *n* = 2 rats. For comparison, LFP examples and average autocorrelation are plotted for the same rats after 1 mM PTX.
(TIF)

S4 Fig Average MUA-triggered LFP waveforms (black) in the striatum show strong positive coupling under the two drug conditions: PTX (*n* = 4), IEM-1460 (*n* = 4). Gray areas indicate ±3 SD of MUA-shuffled LFP averages.
(TIF)

S5 Fig Average MUA-triggered LFP waveforms (black) in the cortex show strong negative coupling under all conditions: baseline (*n* = 5), PTX (*n* = 5), and IEM-1460 (*n* = 2). Gray areas indicate ±3 SD of MUA-shuffled LFP averages.
(TIF)

S6 Fig nLFP activity in organotypic cultures matures after ~2 wk in vitro. Average normalized power spectral density (PSD) of cortical and striatal nLFPs at 6, 14, and 21 DIV (from left to right; shaded area indicates the standard error). Data for this figure are in S7 Data.
(TIF)

S7 Fig Reduction of cortical and striatal inhibition results in similar dynamics as measured by the distribution of spatiotemporal cluster sizes. (A) Discrete (left) and continuous (right) spatiotemporal cluster size distributions for cortex and striatum in the presence of 4 μM PTX in the culture medium. Note the increase in the probability of larger spatiotemporal clusters for both cortex and striatum (see also Fig 3H). Vertical arrow indicates system size for the cortical sub-array (31 electrodes). (B) D_KS_ between cortical and striatal cluster size distributions (*n* = 8) under baseline, PTX, and 24-h washout condition. rANOVA, F(2,14) = 11.67, *p* < 0.001, Bonferroni: **p < 0.01. Data for this figure are in S7 Data.
(TIF)

S8 Fig Two-compartment chamber for locally confined drug application in the striatum as confirmed by dye staining with sulforhodamine 101 (SR101). SR101 stains glia cells, which are present in cortex and striatum. (A) Brightfield image of a cortex-striatum-substantia nigra culture (DIV 19) showing the cortex (ctx) and striatum (str), the coverslip (cv, approximately 300 μm above tissue) for bath compartmentalization, and the pipette (p) for dye application. The gaps between coverslip and chamber were sealed with agar pieces (not visible in picture). The white dashed line shows the approximate border between cortex and striatum. The white and black squares show approximate locations of the imaging regions for cortex and striatum, respectively. ACSF flow was from cortex to striatum. SR101 ejection as indicated in figure panels. Scale bar: 200 μm. (B) Left: No SR101 ejection showing minimal autofluorescence under the given imaging conditions. Inset shows the same image with increased gain for comparison with C. (B) Right: Local striatal ejection of SR101 (200 μM, 5 min at 15 μl/min) stained glia cells in the striatal but not the cortical compartment. Scale bar: 100 μm. (C) Top: Local application of diluted SR101 (1 μM, 5 min at 15 μl/min) in the cortical compartment weakly increased fluorescence and labeled previously unstained glia cells and presumable processes (inset). Same scale as in B. (C) Bottom: Probability density function (PDF) of the fluorescence intensity in the cortical region. Focal application of diluted SR101 in the cortex (1 μM, ctx) led to a significant increase in fluorescence (blue line). Control condition and ejection of 200 μM SR101 in the striatal compartment resulted in almost identical PDFs (gray and red-dashed lines, respectively), indicating negligible spillover from striatal to cortical compartment.
(TIF)

S9 Fig Intrastriatal inhibition and not midbrain input is responsible for the observed striatal dynamics under normal conditions. (A) Acute substantia nigra lesion with post-hoc TH-immunostaining (Str, striatum; SNc, substantia nigra pars compacta; scale bar: 200 μm). Seen are a dense cluster of TH-positive neurons in the SNc and a dense network of TH-positive fibers in the striatum with the acute lesion between the two structures marked by a white, dotted line. (B) Average ΔF/F peak amplitudes during baseline, PTX, and after washout (*n* = 4, rANOVA, F(2,12) = 46.9, *p* < 0.001, Bonferroni: ***p < 0.001) show a similar profile as for the condition without acute midbrain lesion. Data for this figure are in S7 Data.
(TIF)

S1 Table List of rats used for in vivo experiments. Symbols and their meanings: ✓ recorded/observed,—not recorded, × not observed, (*) strong orofacial movements (not analyzed).
(XLSX)

S1 Movie Example of PTX-induced involuntary movements in the left front paw in response to drug injection into the right striatum. The movement signal was extracted from a region of interest (ROI, white square) around the left front paw.
(AVI)

S2 Movie Example of IEM-1460-induced involuntary movements in the left front paw in response to drug injection into the right striatum. The movement signal was extracted from a region of interest (ROI, white square) around the left front paw.
(AVI)

S1 Data Data for Fig 1. (XLSX)

S2 Data Data for Fig 2. (XLSX)

S3 Data Data for Fig 3. (XLSX)

S4 Data Data for Fig 4. (XLSX)

S5 Data Data for Fig 5. (XLSX)

S6 Data Data for Fig 6. (XLSX)

S7 Data Data for S6, S7 and S9 Figs. (XLSX)

## Abbreviations

CC: cross-correlation
DIV: days in vitro
D_KS_: Kolmogorov–Smirnov distance
GABA: γ-aminobutyric acid
GluR2: glutamate receptor 2
IN: interneuron
KS: Kolmogorov–Smirnov
LFP: local field potential
LLR: log-likelihood ratio
MEA: microelectrode array
MUA: multi-unit activity
MWA: microwire array
nLFP: negative LFP
NMDA: N-methyl-D-aspartate
OGB: Oregon Green 488 BAPTA-1
pLFP: positive LFP
PDF: probability density function
PTX: picrotoxin
SD: standard deviation
SPN: spiny projection neuron
TH: tyrosine-hydroxylase

## References

[1] Haimovici A, Tagliazucchi E, Balenzuela P, Chialvo DR. Brain organization into resting state networks emerges at criticality on a model of the human connectome. Phys Rev Lett 2013; 110: 178101 10.1103/PhysRevLett.110.178101 23679783

[2] Tagliazucchi E, Balenzuela P, Fraiman D, Chialvo DR. Criticality in large-scale brain fMRI dynamics unveiled by a novel point process analysis. Front Physiol 2012; 3(15). 10.3389/fphys.2012.00015 PMC3274757 22347863

[3] Linkenkaer-Hansen K, Nikouline VV, Palva JM, Ilmoniemi RJ. Long-range temporal correlations and scaling behavior in human brain oscillations. J Neurosci 2001; 21: 1370–1377. 11160408 10.1523/JNEUROSCI.21-04-01370.2001 PMC6762238

[4] Beggs JM, Plenz D. Neuronal avalanches in neocortical circuits. J Neurosci 2003; 23: 11167–11177. 14657176 10.1523/JNEUROSCI.23-35-11167.2003 PMC6741045

[5] Stewart CV, Plenz D. Inverted-U profile of dopamine-NMDA-mediated spontaneous avalanche recurrence in superficial layers of rat prefrontal cortex. J Neurosci 2006; 26: 8148–8159. 10.1523/JNEUROSCI.0723-06.2006 16885228 PMC6673780

[6] Pasquale V, Massobrio P, Bologna LL, Chiappalone M, Martinoia S. Self-organization and neuronal avalanches in networks of dissociated cortical neurons. Neurosci 2008; 153: 1354–1369. 10.1109/EMBC.2015.7319452. 18448256

[7] Bellay T, Klaus A, Seshadri S, Plenz D. Irregular spiking of pyramidal neurons organizes as scale-invariant neuronal avalanches in the awake state. eLife 2015; 4: e07224 10.7554/eLife.07224 26151674 PMC4492006

[8] Gireesh ED, Plenz D. Neuronal avalanches organize as nested theta- and beta/gamma-oscillations during development of cortical layer 2/3. Proc Natl Acad Sci U S A 2008; 105: 7576–7581. 10.1073/pnas.0800537105 18499802 PMC2396689

[9] Scott G, Fagerholm ED, Mutoh H, Leech R, Sharp DJ, et al Voltage imaging of waking mouse cortex reveals emergence of critical neuronal dynamics. J Neurosci 2014; 34: 16611–16620. 10.1523/JNEUROSCI.3474-14.2014 25505314 PMC4261090

[10] Petermann T, Thiagarajan T, Lebedev MA, Nicolelis MA, Chialvo DR, et al Spontaneous cortical activity in awake monkeys composed of neuronal avalanches. Proc Natl Acad Sci U S A 2009; 106: 15921–15926. 10.1073/pnas.0904089106 19717463 PMC2732708

[11] Yu S, Klaus A, Yang H, Plenz D. Scale-invariant neuronal avalanche dynamics and the cut-off in size distributions. PLoS ONE 2014; 9: e99761 10.1371/journal.pone.0099761 24927158 PMC4057403

[12] Yu S, Yang H, Shriki O, Plenz D. Universal organization of resting brain activity at the thermodynamic critical point. Front Sys Neurosci 2013; 7(42). 10.3389/fnsys.2013.00042 PMC3749752 23986660

[13] Yu S, Yang H, Nakahara H, Santos GS, Nikolic D, et al Higher-order interactions characterized in cortical activity. J Neurosci 2011; 31: 17514–17526. 10.1523/JNEUROSCI.3127-11.2011 22131413 PMC6623824

[14] Shriki O, Alstott J, Carver F, Holroyd T, Henson RN, et al Neuronal avalanches in the resting MEG of the human brain. J Neurosci 2013; 33: 7079–7090. 10.1523/JNEUROSCI.4286-12.2013 23595765 PMC3665287

[15] Palva JM, Zhigalov A, Hirvonen J, Korhonen O, Linkenkaer-Hansen K, et al Neuronal long-range temporal correlations and avalanche dynamics are correlated with behavioral scaling laws. Proc Natl Acad Sci U S A 2013; 110: 3585–3590. 10.1073/pnas.1216855110 23401536 PMC3587255

[16] Plenz D, Thiagarajan TC. The organizing principles of neuronal avalanches: cell assemblies in the cortex? Trends Neurosci 2007; 30: 101–110. 10.1016/j.tins.2007.01.005 17275102

[17] Chialvo DR. Emergent complex neural dynamics. Nat Phys 2010; 6: 744–750.

[18] Shew WL, Plenz D. The functional benefits of criticality in the cortex. Neuroscientist 2013; 19: 88–100. 10.1177/1073858412445487 22627091

[19] Shew WL, Yang H, Yu S, Roy R, Plenz D. Information capacity is maximized in balanced cortical networks with neuronal avalanches. J Neurosci 2011; 5: 55–63. 10.1523/JNEUROSCI.4637-10.2011 21209189 PMC3082868

[20] Yang H, Shew WL, Roy R, Plenz D. Maximal variability of phase synchrony in cortical networks with neuronal avalanches. J Neurosci 2012; 32: 1061–1072. 10.1523/JNEUROSCI.2771-11.2012 22262904 PMC3319677

[21] Alexander GE, Crutcher MD, DeLong MR. Basal Ganglia-Thalamocortical Circuits—Parallel Substrates for Motor, Oculomotor, Prefrontal and Limbic Functions. Prog Brain Res 1990; 85: 119–146. 2094891

[22] Albin RL, Young AB, Penney JB. The functional anatomy of basal ganglia disorders. Trends Neurosci 1989; 12: 366–375. 2479133 10.1016/0166-2236(89)90074-x

[23] Mink JW. The basal ganglia and involuntary movements: Impaired inhibition of competing motor patterns. Arch Neurol 2003; 60: 1365–1368. 10.1001/archneur.60.10.1365 14568805

[24] Jin X, Costa RM. Start/stop signals emerge in nigrostriatal circuits during sequence learning. Nature 2010; 466: 457–462. 10.1038/nature09263 20651684 PMC3477867

[25] Bronfeld M, Bar-Gad I. Tic disorders: what happens in the basal ganglia? Neuroscientist 2013; 19: 101–108. 10.1177/1073858412444466 22596263

[26] Kawaguchi Y, Wilson CJ, Augood SJ, Emson PC. Striatal interneurons: chemical, physiological and morphological characterization. Trends Neurosci 1995; 18: 527–535. 8638293 10.1016/0166-2236(95)98374-8

[27] Oorschot DE. Cell types in the different nuclei of the basal ganglia In: Steiner H, Tseng KY, editors. Handbook of basal ganglia structure and function. London: Academic Press/Elsevier pp. 63–74; 2010.

[28] Czubayko U, Plenz D. Fast synaptic transmission between striatal spiny projection neurons. Proc Natl Acad Sci U S A 2002; 99: 15764–15769. 10.1073/pnas.242428599 12438690 PMC137790

[29] Tunstall MJ, Oorschot DE, Kean A, Wickens JR. Inhibitory interactions between spiny projection neurons in the rat striatum. J Neurophysiol 2002; 88: 1263–1269. 12205147 10.1152/jn.2002.88.3.1263

[30] Koos T, Tepper JM, Wilson CJ. Comparison of IPSCs evoked by spiny and fast-spiking neurons in the neostriatum. J Neurosci 2004; 24: 7916–7922. 10.1523/JNEUROSCI.2163-04.2004 15356204 PMC6729926

[31] Groves PM. A theory of the functional organization of the neostriatum and the neostriatal control of voluntary movement. Brain Research (Amsterdam) 1983; 5: 109–132. 10.1016/0165-0173(83)90011-5 6131733

[32] Ponzi A, Wickens J. Sequentially switching cell assemblies in random inhibitory networks of spiking neurons in the striatum. J Neurosci 2010; 30: 5894–5911. 10.1523/JNEUROSCI.5540-09.2010 20427650 PMC6632589

[33] Wiechert MT, Judkewitz B, Riecke H, Friedrich RW. Mechanisms of pattern decorrelation by recurrent neuronal circuits. Nat Neurosci 2010; 13: 1003–1010. 10.1038/nn.2591 20581841

[34] Koos T, Tepper JM. Inhibitory control of neostriatal projection neurons by GABAergic interneurons. Nat Neurosci 1999; 2: 467–472. 10.1038/8138 10321252

[35] Gustafson N, Gireesh-Dharmaraj E, Czubayko U, Blackwell KT, Plenz D. A comparative voltage and current-clamp analysis of feedback and feedforward synaptic transmission in the striatal microcircuit in vitro. J Neurophysiol 2006; 95: 737–752. 10.1152/jn.00802.2005 16236782

[36] Planert H, Szydlowski SN, Hjorth JJ, Grillner S, Silberberg G. Dynamics of synaptic transmission between fast-spiking interneurons and striatal projection neurons of the direct and indirect pathways. J Neurosci 2010; 30: 3499–3507. 10.1523/JNEUROSCI.5139-09.2010 20203210 PMC6634087

[37] Klaus A, Planert H, Hjorth JJ, Berke JD, Silberberg G, et al Striatal fast-spiking interneurons: from firing patterns to postsynaptic impact. Front Syst Neurosci 2011; 5: 1–17; 21808608 10.3389/fnsys.2011.00057 PMC3139213

[38] Gittis AH, Leventhal DK, Fensterheim BA, Pettibone JR, Berke JD, et al Selective inhibition of striatal fast-spiking interneurons causes dyskinesias. J Neurosci 2011; 31: 15727–15731. 10.1523/JNEUROSCI.3875-11.2011 22049415 PMC3226784

[39] Kalanithi PSA, Zheng W, Kataoka Y, DiFiglia M, Grantz H, et al Altered parvalbumin-positive neuron distribution in basal ganglia of individuals with Tourette syndrome. Proc Natl Acad Sci U S A 2005; 102: 13307–13312. 10.1073/pnas.0502624102 16131542 PMC1201574

[40] Kataoka Y, Kalanithi PSA, Grantz H, Schwartz ML, Saper C, et al Decreased number of parvalbumin and cholinergic interneurons in the striatum of individuals with Tourette syndrome. J Comp Neurol 2010; 518: 277–291. 10.1002/cne.22206 19941350 PMC2846837

[41] Tarsy D, Pycock C, Meldrum B, Marsden C. Focal contralateral myoclonus produced by inhibition of GABA action in the caudate nucleus of rats. Brain 1978; 101: 143–162. 638722 10.1093/brain/101.1.143

[42] Patel S, Slater P. Analysis of the brain regions involved in myoclonus produced by intracerebral picrotoxin. Neuroscience 1987; 20: 687–693. 3587612 10.1016/0306-4522(87)90119-9

[43] Yoshida M, Nagatsuka Y, Muramatsu S, Niijima K. Differential roles of the caudate nucleus and putamen in motor behavior of the cat as investigated by local injection of GABA antagonists. Neurosci Res 1991; 10: 34–51. 1851976 10.1016/0168-0102(91)90018-t

[44] Bronfeld M, Yael D, Belelovsky K, Bar-Gad I. Motor tics evoked by striatal disinhibition in the rat. Front Syst Neurosci 2013; 7(50). 10.3389/fnsys.2013.00050 PMC3776161 24065893

[45] Israelashvili M, Bar-Gad I. Corticostriatal Divergent Function in Determining the Temporal and Spatial Properties of Motor Tics. J Neurosci 2015; 35: 16340–16351. 10.1523/JNEUROSCI.2770-15.2015 26674861 PMC4679818

[46] Buldakova SL, Kim KK, Tikhonov DB, Magazanik LG. Selective blockade of Ca2+ permeable AMPA receptors in CA1 area of rat hippocampus. Neuroscience 2007; 144: 88–99. 10.1016/j.neuroscience.2006.09.005 17097234

[47] Deng YP, Xie JP, Wang HB, Lei WL, Chen Q, et al Differential localization of the GluR1 and GluR2 subunits of the AMPA-type glutamate receptor among striatal neuron types in rats. J Chem Neuroanat 2007; 33: 167–192. 10.1016/j.jchemneu.2007.02.008 17446041 PMC1993922

[48] Muramatsu S, Yoshida M, Nakamura S. Electrophysiological study of dyskinesia produced by microinjection of picrotoxin into the striatum of the rat. Neuroscience 1990; Research 7: 369–380. 10.1016/0168-0102(90)90011-3 2156198

[49] Shew WL, Yang H, Petermann T, Roy R, Plenz D. Neuronal avalanches imply maximum dynamic range in cortical networks at criticality. J Neurosci 2009; 29: 15595–15600. 10.1523/JNEUROSCI.3864-09.2009 20007483 PMC3862241

[50] Klaus A, Yu S, Plenz D. Statistical analyses support power law distributions found in neuronal avalanches. PLoS ONE 2011; 6: e19779 10.1371/journal.pone.0019779 21720544 PMC3102672

[51] Plenz D, Kitai ST. 'Up' and 'down' states in striatal medium spiny neurons simultaneously recorded with spontaneous activity in fast-spiking interneurons studied in cortex-striatum-substantia nigra organotypic cultures. J Neurosci 1998; 18: 266–283. 9412506 10.1523/JNEUROSCI.18-01-00266.1998 PMC6793428

[52] Plenz D, Stewart CV, Shew W, Yang H, Klaus A, et al Multi-electrode array recordings of neuronal avalanches in organotypic cultures. J Vis Exp 2011; e2949 10.3791/2949 PMC3211128 21841767

[53] Plenz D, Kitai ST. Organotypic cortex-striatum-mesencephalon cultures: the nigro-striatal pathway. Neurosci Lett 1996; 209: 177–180. 8736639 10.1016/0304-3940(96)12644-6

[54] Stewart CV, Plenz D. Homeostasis of neuronal avalanches during postnatal cortex development in vitro. J Neurosci Meth 2007; 169: 405–416. 18082894 10.1016/j.jneumeth.2007.10.021 PMC2743406

[55] Belić JJ, Klaus A, Plenz D, Hellgren Kotaleski J. Mapping of Cortical Avalanches to the Striatum In: Liljenström H, editor. Advances in Cognitive Neurodynamics (IV): Proceedings of the Fourth International Conference on Cognitive Neurodynamics—2013. Dordrecht: Springer Netherlands 2015; pp. 291–297.

[56] Kerr JN, Plenz D. Action potential timing determines dendritic calcium during striatal up-states. J Neurosci 2004; 24: 877–885. 10.1523/JNEUROSCI.4475-03.2004 14749432 PMC6729828

[57] Kerr JN, Plenz D. Dendritic calcium encodes striatal neuron output during Up-states. J Neurosci 2002; 22: 1499–1512. 10.1523/JNEUROSCI.4475-03.2004 11880480 PMC6758904

[58] Plenz D, Aertsen A. Neuronal dynamics in cortex-striatum co-cultures. II. Spatio-temporal characteristics of neuronal activity. Neuroscience 1996; 70: 893–924. 8848173 10.1016/0306-4522(95)00405-x

[59] Costa RM, Lin S-C, Sotnikova TD, Cyr M, Gainetdinov RR, et al Rapid alterations in corticostriatal ensemble coordination during acute dopamine-dependent motor dysfunction. Neuron 2006; 52: 359–369. 10.1016/j.neuron.2006.07.030 17046697

[60] Oldenburg Ian A, Sabatini Bernardo L. Antagonistic but Not Symmetric Regulation of Primary Motor Cortex by Basal Ganglia Direct and Indirect Pathways. Neuron 2015; 86: 1174–1181. 10.1016/j.neuron.2015.05.008 26050037 PMC4458709

[61] Kincaid AE, Zheng T, Wilson CJ. Connectivity and convergence of single corticostriatal axons. J Neurosci 1998; 18: 4722–4731. 9614246 10.1523/JNEUROSCI.18-12-04722.1998 PMC6792707

[62] Zheng T, Wilson CJ. Corticostriatal combinatorics: the implications of corticostriatal axonal arborizations. J Neurophysiol 2002; 87: 1007–1017. 11826064 10.1152/jn.00519.2001

[63] Smith Y, Raju D, Nanda B, Pare J-F, Galvan A, et al The thalamostriatal systems: anatomical and functional organization in normal and parkinsonian states. Brain Res Bull 2009; 78: 60–68. 10.1016/j.brainresbull.2008.08.015 18805468 PMC2656644

[64] Mallet N, Micklem BR, Henny P, Brown MT, Williams C, et al Dichotomous organization of the external globus pallidus. Neuron 2012; 74: 1075–1086. 10.1016/j.neuron.2012.04.027 22726837 PMC3407962

[65] Tritsch NX, Ding JB, Sabatini BL. Dopaminergic neurons inhibit striatal output through non-canonical release of GABA. Nature 2012; 490: 262–266. 10.1038/nature11466 23034651 PMC3944587

[66] Bolshakov KV, Kim KH, Potapjeva NN, Gmiro VE, Tikhonov DB, et al Design of antagonists for NMDA and AMPA receptors. Neuropharmacology 2005; 49: 144–155. 10.1016/j.neuropharm.2005.02.007 15996563

[67] English DF, Ibanez-Sandoval O, Stark E, Tecuapetla F, Buzsaki G, et al GABAergic circuits mediate the reinforcement-related signals of striatal cholinergic interneurons. Nat Neurosci 2012; 15: 123–130. 10.1038/nn.2984 PMC3245803 22158514

[68] Nelson AB, Hammack N, Yang CF, Shah NM, Seal RP, et al Striatal cholinergic interneurons Drive GABA release from dopamine terminals. Neuron 2014; 82: 63–70. 10.1016/j.neuron.2014.01.023 24613418 PMC3976769

[69] Aoki S, Liu AW, Zucca A, Zucca S, Wickens JR. Role of Striatal Cholinergic Interneurons in Set-Shifting in the Rat. J Neurosci 2015; 35: 9424–9431. 10.1523/JNEUROSCI.0490-15.2015 26109665 PMC6605199

[70] Adler A, Katabi S, Finkes I, Prut Y, Bergman H. Different correlation patterns of cholinergic and GABAergic interneurons with striatal projection neurons. Front Syst Neurosci 2013; 7;(47). 10.3389/fnsys.2013.00047 PMC3760072 24027501

[71] Rock C, Zurita H, Wilson C, junior Apicella A. An inhibitory corticostriatal pathway. eLife 2016; 5: e15890 10.7554/eLife.15890 27159237 PMC4905740

[72] Kravitz AV, Freeze BS, Parker PR, Kay K, Thwin MT, et al Regulation of parkinsonian motor behaviours by optogenetic control of basal ganglia circuitry. Nature 2010; 466: 622–626. 10.1038/nature09159 20613723 PMC3552484

[73] Stachniak TJ, Ghosh A, Sternson SM. Chemogenetic synaptic silencing of neural circuits localizes a hypothalamus→ midbrain pathway for feeding behavior. Neuron 2014; 82: 797–808. 10.1016/j.neuron.2014.04.008 24768300 PMC4306349

[74] Lin JY, Sann SB, Zhou K, Nabavi S, Proulx CD, et al Optogenetic inhibition of synaptic release with chromophore-assisted light inactivation (CALI). Neuron 2013; 79: 241–253. 10.1016/j.neuron.2013.05.022 23889931 PMC3804158

[75] Berke JD. Uncoordinated Firing Rate Changes of Striatal Fast-Spiking Interneurons during Behavioral Task Performance. J Neurosci 2008; 28: 10075–10080. 10.1523/JNEUROSCI.2192-08.2008 18829965 PMC2613805

[76] Joshua M, Adler A, Mitelman R, Vaadia E, Bergman H. Midbrain Dopaminergic Neurons and Striatal Cholinergic Interneurons Encode the Difference between Reward and Aversive Events at Different Epochs of Probabilistic Classical Conditioning Trials. J Neurosci 2008; 28: 11673–11684. 10.1523/JNEUROSCI.3839-08.2008 18987203 PMC6671303

[77] López-Huerta VG, Carrillo-Reid L, Galarraga E, Tapia D, Fiordelisio T, et al The Balance of Striatal Feedback Transmission Is Disrupted in a Model of Parkinsonism. J Neurosci 2013; 33: 4964–4975. 10.1523/JNEUROSCI.4721-12.2013 23486967 PMC6619024

[78] Carrillo-Reid L, Tecuapetla F, Tapia D, Hernández-Cruz A, Galarraga E, et al Encoding network states by striatal cell assemblies. J Neurophysiol 2008; 99: 1435–1450. 10.1152/jn.01131.2007 18184883

[79] Wickens JR, Arbuthnott GW, Shindou T. Simulation of GABA function in the basal ganglia: computational models of GABAergic mechanisms in basal ganglia function. Prog Brain Res 2007; 160: 313–329. 10.1016/S0079-6123(06)60018-6 17499122

[80] Damodaran S, Cressman JR, Jedrzejewski-Szmek Z, Blackwell KT. Desynchronization of Fast-Spiking Interneurons Reduces β-Band Oscillations and Imbalance in Firing in the Dopamine-Depleted Striatum. J Neurosci 2015; 35: 1149–1159. 10.1523/JNEUROSCI.3490-14.2015 25609629 PMC4300321

[81] Ponzi A, Wickens JR. Optimal balance of the striatal medium spiny neuron network. PLoS Comput Biol 2013; 9: e1002954 10.1371/journal.pcbi.1002954 23592954 PMC3623749

[82] Wilson CJ. Active decorrelation in the basal ganglia. Neuroscience 2013; 250: 467–482. 10.1016/j.neuroscience.2013.07.032 23892007 PMC3772785

[83] Wickens JR, Wilson CJ. Regulation of action-potential firing in spiny neurons of the rat neostriatum in vivo. J Neurophysiol 1998; 79: 2358–2364. 9582211 10.1152/jn.1998.79.5.2358

[84] Oorschot DE, Lin N, Cooper BH, Reynolds JNJ, Sun H, et al Synaptic connectivity between rat striatal spiny projection neurons in vivo: unexpected multiple somatic innervation in the context of overall sparse proximal connectivity. Basal Ganglia 2013; 3: 93–108.

[85] Lee HJ, Weitz AJ, Bernal-Casas D, Duffy BA, Choy M, Kravitz AV, Kreitzer AV, Kreitzer AC, Lee JH. Activation of direct and indirect pathway medium spiny neurons drives distinct brain-wide responses. Neuron 2016; 91: 412–424. 27373834 10.1016/j.neuron.2016.06.010 PMC5528162

[86] Gage GJ, Stoetzner CR, Wiltschko AB, Berke JD. Selective activation of striatal fast-spiking interneurons during choice execution. Neuron 2010; 67: 466–479. 10.1016/j.neuron.2010.06.034 20696383 PMC2920892

[87] Gittis AH, Nelson AB, Thwin MT, Palop JJ, Kreitzer AC. Distinct roles of GABAergic interneurons in the regulation of striatal output pathways. J Neurosci 2010; 30: 2223–2234. 10.1523/JNEUROSCI.4870-09.2010 20147549 PMC2836801

[88] Ikegaya Y, Le Bon-Jego M, Yuste R. Large-scale imaging of cortical network activity with calcium indicators. Neurosci Res 2005; 52: 132–138. 10.1016/j.neures.2005.02.004 15893573

[89] Stern EA, Jaeger D, Wilson CJ. Membrane potential synchrony of simultaneously recorded striatal spiny neurons in vivo. Nature 1998; 394: 475–478. 10.1038/28848 9697769

[90] Clauset A, Shalizi CR, Newman MEJ. Power-law distributions in empirical data. SIAM Rev 2009; 51: 42 10.1137/070710111

