# Supplementary figures and images for: A Low-Correlation Resting State of the Striatum during Cortical Avalanches and Its Role in Movement Suppression
Source: PLoS Biol. 2016 Dec 6;14(12):e1002582. doi: 10.1371/journal.pbio.1002582 (PMC5147796; doi:10.1371/journal.pbio.1002582)

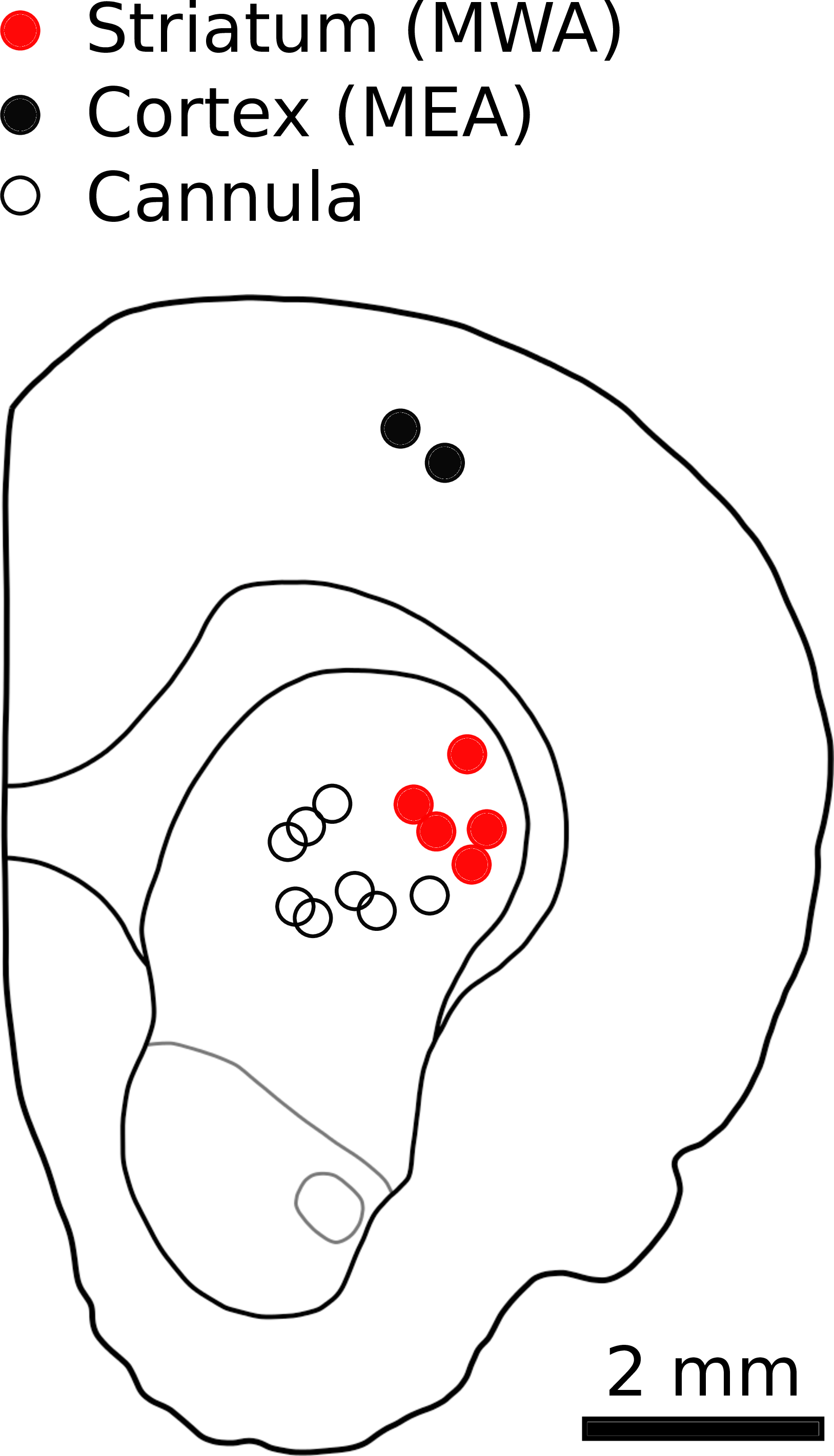

Supplement: S1 Fig — (TIF) [file pbio.1002582.s001.tif]

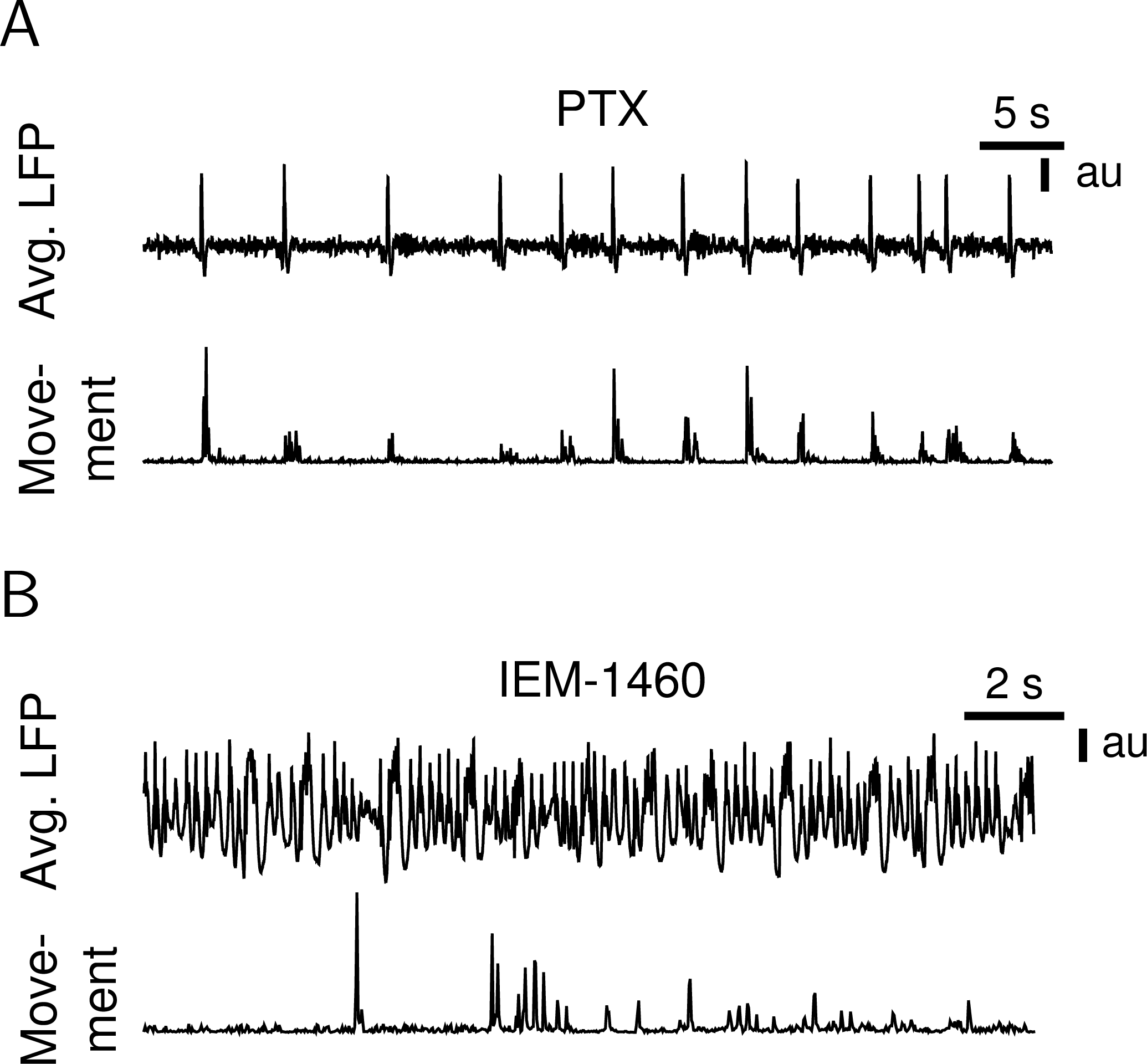

Supplement: S2 Fig — (A) Example of simultaneously recorded LFP (average) and involuntary movements in the contralateral front paw after local striatal injection of PTX (1 mM). (B) The same as in A for a different rat under IEM-1460 (5 mM), showing the intermittency of involuntary movements in the presence of continuous oscillatory striatal LFP activity. Note the different time scales in A and B. For the calculation of the “movement” signal, see Materials and Methods. (TIF) [file pbio.1002582.s002.tif]

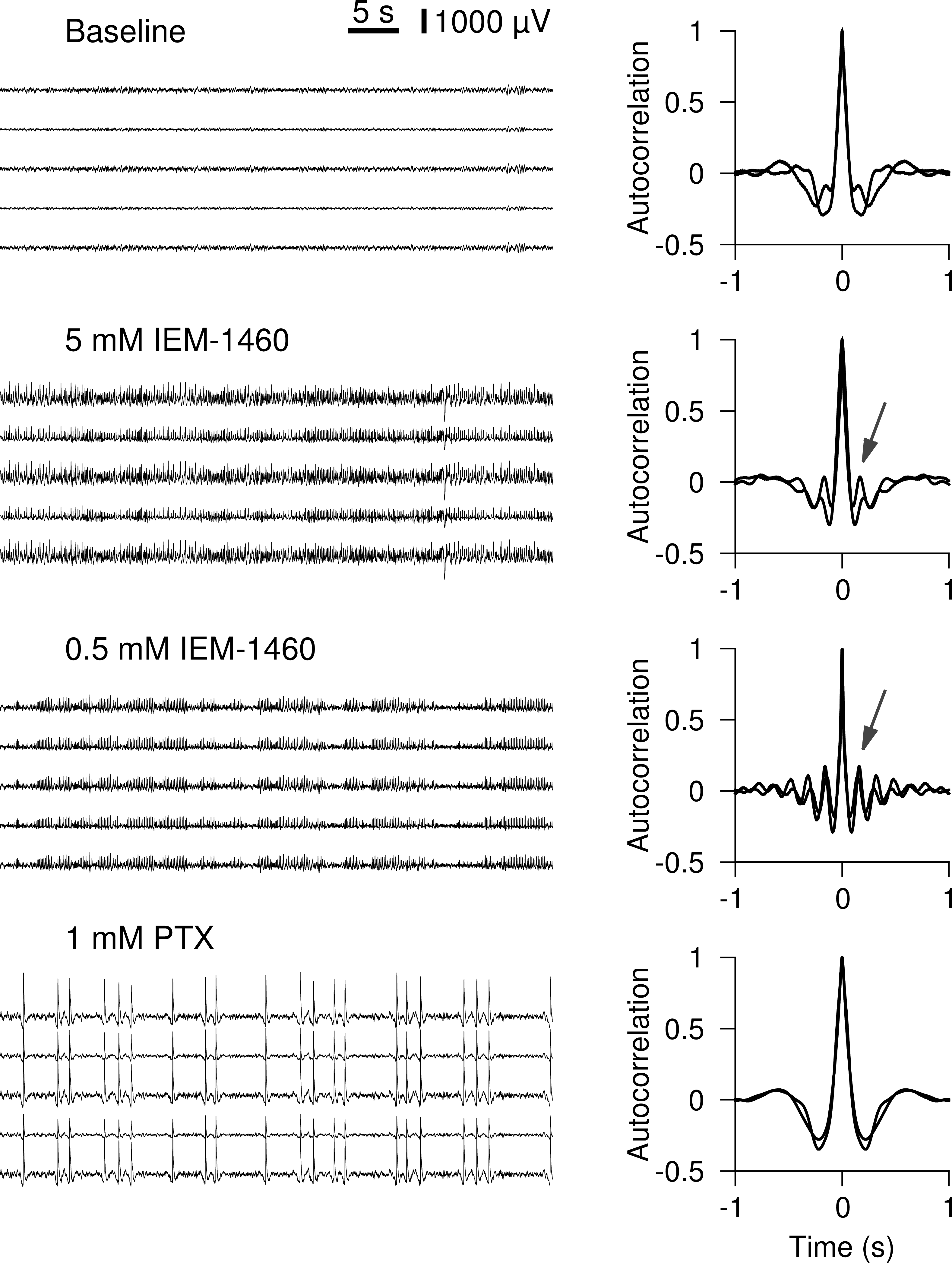

Supplement: S3 Fig — Left column: example LFP traces. Right column: average autocorrelation functions for n = 2 rats. For comparison, LFP examples and average autocorrelation are plotted for the same rats after 1 mM PTX. (TIF) [file pbio.1002582.s003.tif]

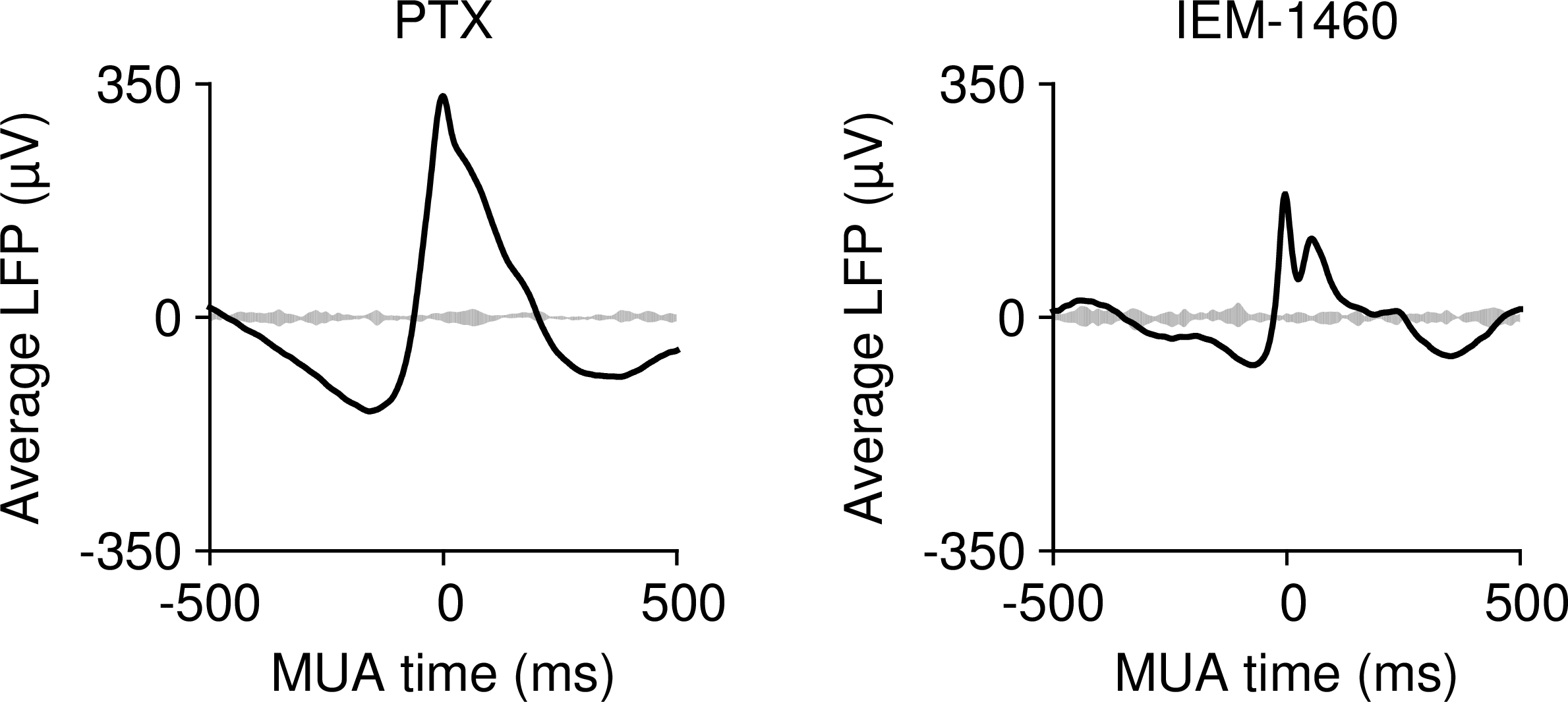

Supplement: S4 Fig — Gray areas indicate ±3 SD of MUA-shuffled LFP averages. (TIF) [file pbio.1002582.s004.tif]

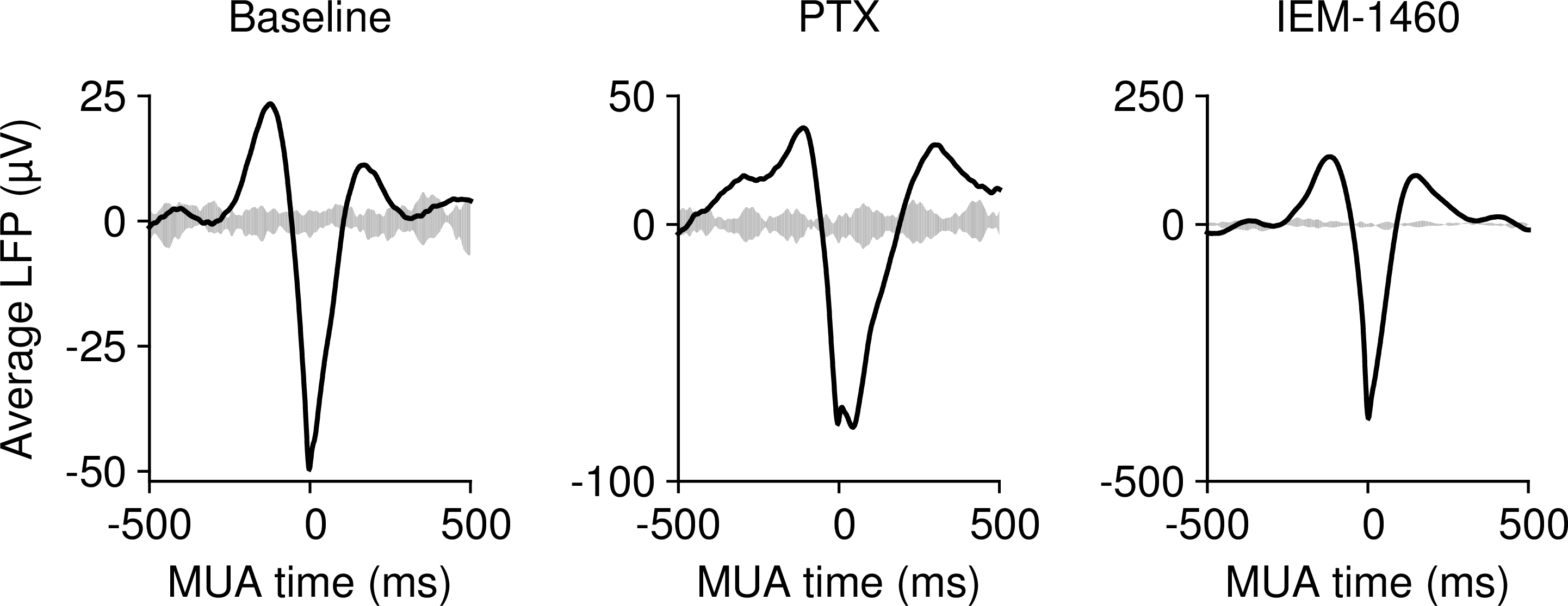

Supplement: S5 Fig — Gray areas indicate ±3 SD of MUA-shuffled LFP averages. (TIF) [file pbio.1002582.s005.tif]

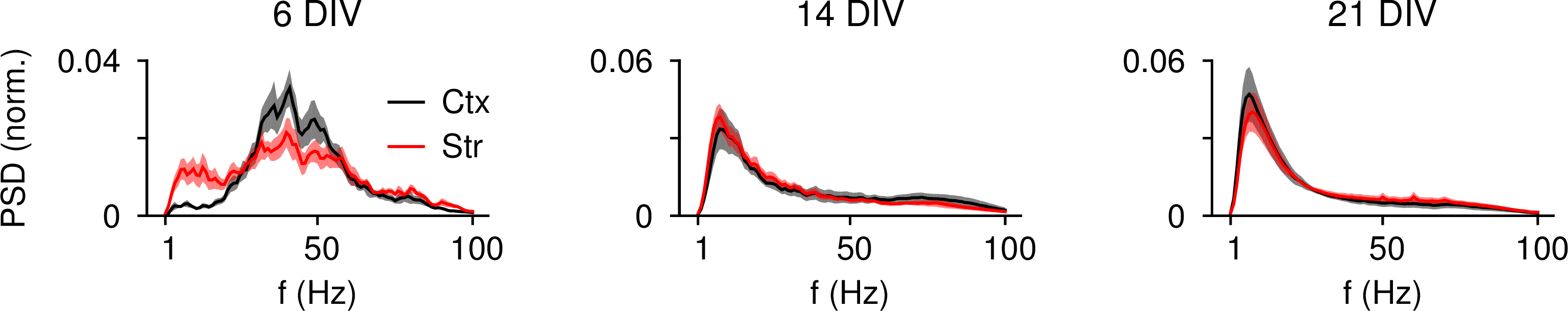

Supplement: S6 Fig — Average normalized power spectral density (PSD) of cortical and striatal nLFPs at 6, 14, and 21 DIV (from left to right; shaded area indicates the standard error). Data for this figure are in S7 Data. (TIF) [file pbio.1002582.s006.tif]

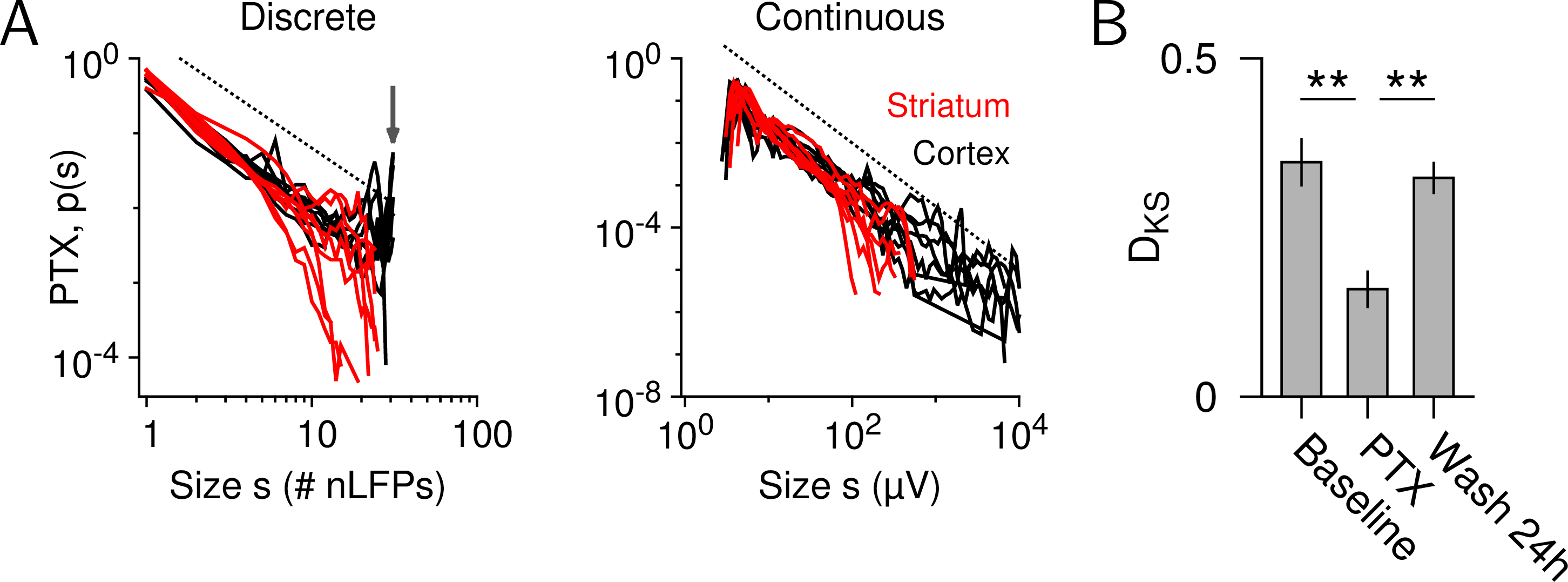

Supplement: S7 Fig — (A) Discrete (left) and continuous (right) spatiotemporal cluster size distributions for cortex and striatum in the presence of 4 μM PTX in the culture medium. Note the increase in the probability of larger spatiotemporal clusters for both cortex and striatum (see also Fig 3H). Vertical arrow indicates system size for the cortical sub-array (31 electrodes). (B) DKS between cortical and striatal cluster size distributions (n = 8) under baseline, PTX, and 24-h washout condition. rANOVA, F(2,14) = 11.67, p < 0.001, Bonferroni: **p < 0.01. Data for this figure are in S7 Data. (TIF) [file pbio.1002582.s007.tif]

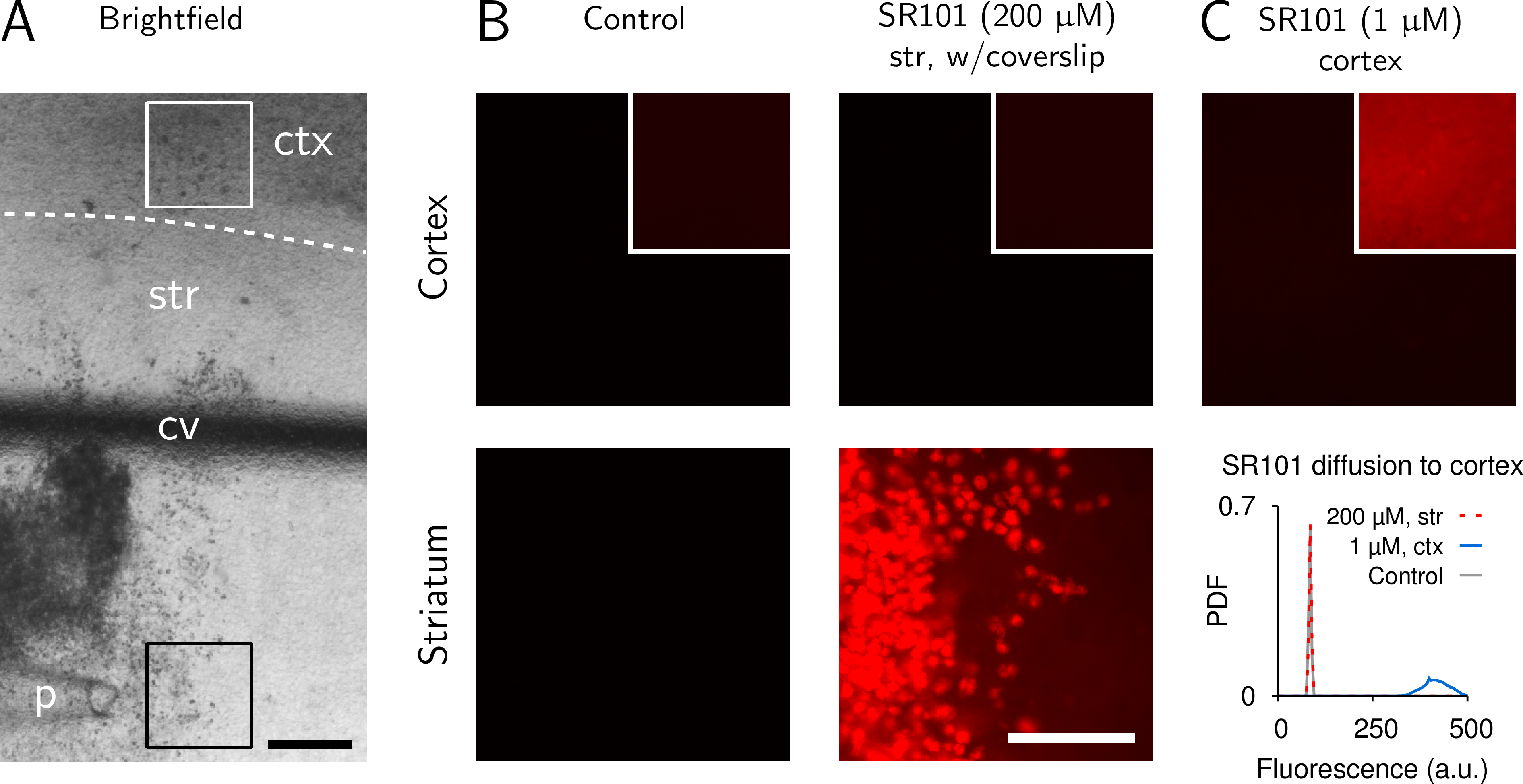

Supplement: S8 Fig — SR101 stains glia cells, which are present in cortex and striatum. (A) Brightfield image of a cortex-striatum-substantia nigra culture (DIV 19) showing the cortex (ctx) and striatum (str), the coverslip (cv, approximately 300 μm above tissue) for bath compartmentalization, and the pipette (p) for dye application. The gaps between coverslip and chamber were sealed with agar pieces (not visible in picture). The white dashed line shows the approximate border between cortex and striatum. The white and black squares show approximate locations of the imaging regions for cortex and striatum, respectively. ACSF flow was from cortex to striatum. SR101 ejection as indicated in figure panels. Scale bar: 200 μm. (B) Left: No SR101 ejection showing minimal autofluorescence under the given imaging conditions. Inset shows the same image with increased gain for comparison with C. (B) Right: Local striatal ejection of SR101 (200 μM, 5 min at 15 μl/min) stained glia cells in the striatal but not the cortical compartment. Scale bar: 100 μm. (C) Top: Local application of diluted SR101 (1 μM, 5 min at 15 μl/min) in the cortical compartment weakly increased fluorescence and labeled previously unstained glia cells and presumable processes (inset). Same scale as in B. (C) Bottom: Probability density function (PDF) of the fluorescence intensity in the cortical region. Focal application of diluted SR101 in the cortex (1 μM, ctx) led to a significant increase in fluorescence (blue line). Control condition and ejection of 200 μM SR101 in the striatal compartment resulted in almost identical PDFs (gray and red-dashed lines, respectively), indicating negligible spillover from striatal to cortical compartment. (TIF) [file pbio.1002582.s008.tif]

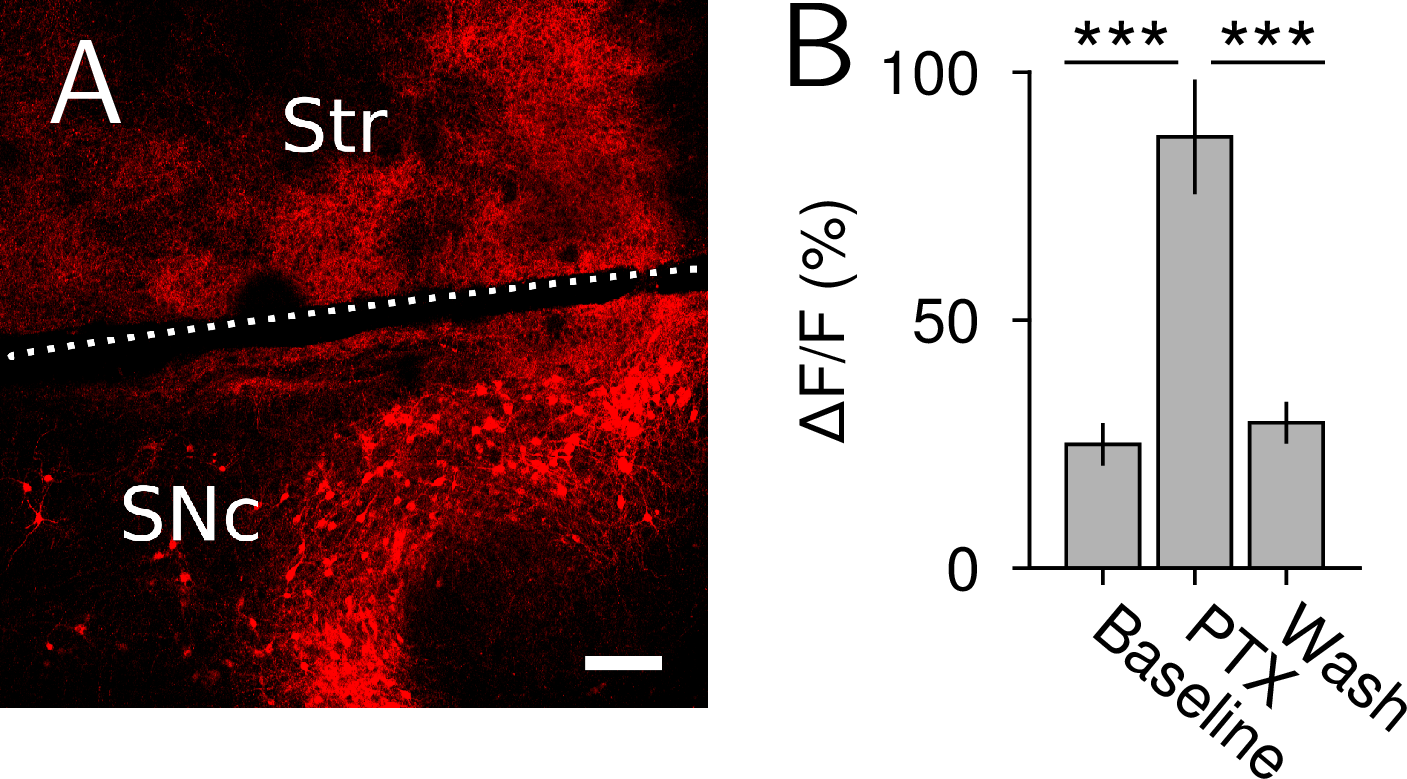

Supplement: S9 Fig — (A) Acute substantia nigra lesion with post-hoc TH-immunostaining (Str, striatum; SNc, substantia nigra pars compacta; scale bar: 200 μm). Seen are a dense cluster of TH-positive neurons in the SNc and a dense network of TH-positive fibers in the striatum with the acute lesion between the two structures marked by a white, dotted line. (B) Average ΔF/F peak amplitudes during baseline, PTX, and after washout (n = 4, rANOVA, F(2,12) = 46.9, p < 0.001, Bonferroni: ***p < 0.001) show a similar profile as for the condition without acute midbrain lesion. Data for this figure are in S7 Data. (TIF) [file pbio.1002582.s009.tif]
